# scNanoATAC-seq: a long-read single-cell ATAC sequencing method to detect chromatin accessibility and genetic variants simultaneously within an individual cell

**DOI:** 10.1038/s41422-022-00730-x

**Published:** 2022-10-11

**Authors:** Yuqiong Hu, Zhenhuan Jiang, Kexuan Chen, Zhangxian Zhou, Xin Zhou, Yan Wang, Jingwei Yang, Bo Zhang, Lu Wen, Fuchou Tang

**Affiliations:** 1 Biomedical Pioneering Innovation Center, School of Life Sciences, Peking University, Beijing, China; 2 Beijing Advanced Innovation Center for Genomics (ICG), Ministry of Education Key Laboratory of Cell Proliferation and Differentiation, Beijing, China; 3 PKU-Tsinghua-NIBS Graduate Program, Academy for Advanced Interdisciplinary Studies, Peking University, Beijing, China; 4 Peking-Tsinghua Center for Life Sciences, Academy for Advanced Interdisciplinary Studies, Peking University, Beijing, China; 5 Department of General Surgery, Third Hospital, Peking University, Beijing, China

**Keywords:** Chromatin analysis, Genomic analysis

Dear Editor,

The accessible chromatin regions were enriched in regulatory elements in human genome and many genetic variations associated with human diseases were identified within them.^1^ Single-cell assay for transposase-accessible chromatin using sequencing (scATAC-seq) on the next-generation sequencing (NGS) platform is a well-established method to detect open chromatin regions within an individual cell.^2^ However, there remain challenges in detection of large-scale structural variations (SVs, including insertions, deletions, duplications, inversions and translocations) and haplotype phasing from scATAC-seq data, which can be well resolved by third-generation sequencing (TGS) platform-based single-molecule long-read sequencing. To integrate advantages of long-read sequencing into scATAC-seq, we developed single-cell assay for transposase‐accessible chromatin on Nanopore sequencing platform (scNanoATAC-seq), a plate-based scATAC-seq method suitable for TGS platform (Supplementary information, Fig. S1).

To evaluate the performance of scNanoATAC-seq on capturing chromatin accessibility in individual cells, we tested the scNanoATAC-seq method in five human cell lines (GM12878, eHAP1, HEK293T, HFF-1 and K562) as well as in human peripheral blood mononuclear cells (PBMCs). The medians of read length of our scNanoATAC-seq libraries ranged from 4000 bp to 4900 bp. We evaluated the quality of the data produced by our method using transcriptional start site (TSS) enrichment, fragment numbers and fractions of read ends in peaks (FRIP) (Supplementary information, Fig. S3e). The scNanoATAC-seq read ends of GM12878 cells showed strong enrichment patterns around TSS and footprints around CCCTC-binding factor (CTCF)-bound sites, which were similar to those in NGS-based scATAC-seq data (Supplementary information, Fig. S2a, b). The pits in the downstream of TSS were proved to be the periodic nucleosome occupancies by the pioneering work of Buenrostro et al. on bulk ATAC-seq.^2^ More nucleosome occupancy pits were detected by our method compared with NGS-based ATAC-seq (Supplementary information, Fig. S2a). Using equivalent numbers of GM12878 cells, 27,290 peaks were overlapped between scNanoATAC-seq and 10× scATAC-seq, accounting for 54.9% of scNanoATAC-seq peaks and 46.2% of 10× scATAC-seq peaks. 42,189 peaks were overlapped with bulk NGS ATAC-seq, accounting for 84.9% of scNanoATAC-seq data. As a comparison, 54,203 peaks of 10× scATAC-seq were overlapped with bulk NGS ATAC-seq, accounting for 91.8% of 10× scATAC-seq peaks. These results indicated comparable performance of these two methods for identifying accessible chromatin regions within an individual cell (Supplementary information, Fig. S2c). The same comparison was made in other cell lines such as K562 cells and HEK293T cells (Supplementary information, Fig. S3d). The chromatin accessibility patterns around marker genes of GM12878 cells (*CD19* and *CD79A*) were comparable between long-read and short-read scATAC-seq (Supplementary information, Fig. S2d). We exploited ENCODE candidate *cis*-regulatory elements (cCREs) to profile distributions of peaks called from NGS-based scATAC-seq data and TGS-based scNanoATAC-seq data of GM12878 cells. Among scNanoATAC-seq peaks, we found that the proportions of CTCF-only elements and distal enhancer-like signatures (dELS) increased, while the proportions of promoter-like signatures (PLS) and proximal enhancer-like signatures (pELS) were relatively lower than NGS-based scATAC-seq (Supplementary information, Fig. S2e), similar to the enrichment profile annotated by gene regions (Supplementary information, Fig. S2f). Consistent annotation results were found in peaks of K562 cells and HEK293T cells (Supplementary information, Fig. S3g, h). When we evaluated precision and recall of peak calling using the cCRE set as a benchmark of regulatory elements, the performance was comparable between NGS-based scATAC-seq and TGS-based scNanoATAC-seq (Supplementary information, Fig. S2g).

We performed two batches of species mixing experiments, using four cell lines mixed in equal quantities, including two human cell lines (K562 and GM12878) and two mouse cell lines (mESC and MEF). In two technical replicates, 2.2% (5 out of 223) and 1.6% (4 out of 248) of single cells were identified as doublets (Supplementary information, Fig. S3a), which was acceptable for single-cell chromatin accessibility sequencing. Next, we projected scNanoATAC-seq data of five human cell lines into the uniform manifold approximation and projection (UMAP) space. Each cell line was well distinguished by unsupervised clustering (Supplementary information, Fig. S3b) without significant batch effects. To estimate the optimal throughput of single cells for scNanoATAC-seq, we simulated single-cell data by sampling reads without replacement from pseudo bulks of each cell line to specific numbers of reads per cell. Ten thousand reads per cell (2000 cells per sequencing run) was the maximal throughput of our method (Supplementary information, Fig. S3c). We identified strong footprints over binding sites of cell type-specific transcription factors, which were consistent with those in 10× scATAC-seq data (Supplementary information, Fig. S3f). To evaluate whether our method works for in vivo samples, we performed scNanoATAC-seq on both sorted and unsorted PBMCs from a single donor. CD4^+^ T cells, CD8^+^ T cells, B cells and monocytes were clearly identified in both sorted and unsorted PBMCs without significant batch effects (Supplementary information, Fig. S4a), based on FACS gating and gene accessibility score at the canonical marker genes (Supplementary information, Fig. S5). To compare peak calling quality of scNanoATAC-seq data with that of 10× scATAC-seq data, a benchmark test based on cCREs was also performed for PBMCs (Supplementary information, Fig. S4b, c). High signals on the key marker gene loci of PBMCs were visualized from the data generated by both our method and 10× scATAC-seq method (Supplementary information, Fig. S4d). Taken together, scNanoATAC-seq performed well in identifying different cell populations and revealing key regulatory features of chromatin accessibility of each cell population.

As long-read sequencing has advantages for haplotype phasing, we haplotyped chromatin accessibility of GM12878 (Fig. 1b, c) after proving the fidelity of our method. We validated allele-specific peaks (ASPs) identified in scNanoATAC-seq by short-read bulk ATAC-seq. Chromatin accessibility signals of both methods were haplotyped by phased heterozygous SNPs of GM12878 provided by Genome in a Bottle Consortium (GIAB).^3^ From scNanoATAC-seq data, we found that 0.77% (384 out of 49,688) of total peaks were allele-specific in GM12878. Among them, 22.4% (86 out of 384) of ASPs contained heterozygous SNPs within the peaks, and were verified by short-read bulk ATAC-seq. 90.9% and 88.1% of maternal and paternal peaks got validated, respectively (Supplementary information, Table S1), which proved that the ASPs identified by two distinct technologies were consistent with each other (Supplementary information, Fig. S6b). Since heterozygous SNPs of most studied samples were not phased, we phased known heterozygous SNPs of GM12878 taking advantage of long reads of scNanoATAC-seq. As a result, the median switch error rate of phasing by chromosome was about 1.4% (Supplementary information, Table S1), which had little negative impact on the detection of ASPs. With heterozygous SNPs phased, we identified 94.7% of ASPs to be true in GM12878 (Supplementary information, Table S1). Thus we proved that ASPs on heterozygous loci were precisely detected by scNanoATAC-seq.

**Fig. 1:**
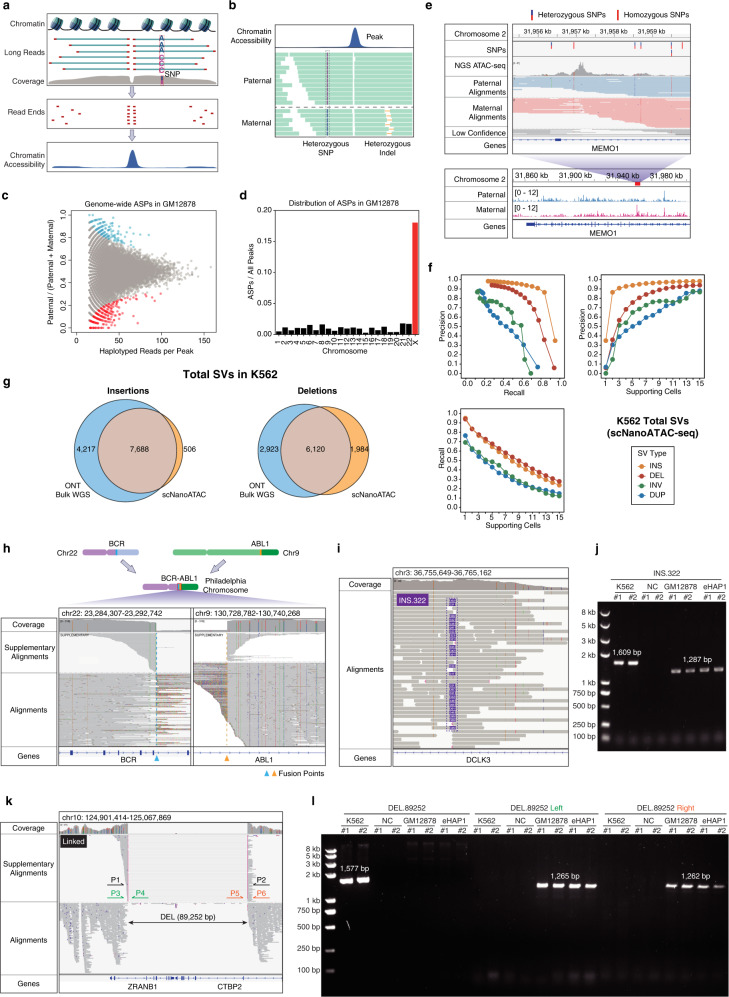
Haplotype phasing and SV detection by scNanoATAC-seq. **a** A schematic diagram of scNanoATAC-seq. **b** A schematic diagram of identifying allele-specific chromatin accessibility by scNanoATAC-seq. Heterozygous SNPs around (outside) rather than inside the peaks were exploited to phase the haplotypes of reads. **c** The proportion of paternal reads and the counts of all haplotyped reads on each peak in GM12878. Significant ASPs (FDR = 0.05) were highlighted in blue (paternal) and red (maternal). **d** Proportions of ASPs in GM12878 on each chromosome. **e** An example of GM12878 ASP inside an intron of *MEMO1* detected by scNanoATAC-seq without any heterozygous SNP inside the peak. Reads were properly haplotyped based on heterozygous SNPs around the ASP. **f** Performance of SV identification in K562 manifested by precision and recall varying with the number of single cells supporting each SV event. **g** A Venn diagram showing the relationships between K562 SV sets called from scNanoATAC-seq and bulk ONT genome sequencing. Insertions and deletions were both supported by at least 5 individual cells. **h** The Philadelphia chromosome causing the *BCR-ABL1* fusion gene was detected by scNanoATAC-seq in K562. **i**, **j** A 322-bp insertion inside an intron of *DCLK3* in K562 was detected by scNanoATAC-seq (**i**) and validated as a somatic mutation by PCR with the GM12878 cell line as a germline control (**j**). Pure water was used for template of PCR as negative control (NC). **k**, **l** A somatic deletion truncating *ZRANB1* and *CTBP2* in K562 was detected by scNanoATAC-seq (**k**), which was validated as a homozygous somatic deletion by PCR products of the deletion region (P1 and P2), the left breakpoint (P3 and P4) and the right breakpoint (P5 and P6) (**l**).

Besides ASPs containing heterozygous SNPs that can be detected by short-read ATAC-seq, there were ASPs without any heterozygous SNP inside them, which were identified by scNanoATAC-seq (Fig. 1e) and supported by evidence of genomic imprinting loci. Those ASPs called by scNanoATAC-seq were located in known imprinted differentially methylated regions (DMRs),^4^ on gene loci of *SNU13* and *TRIM61* (Supplementary information, Fig. S6c, d). The ASP overlapping with the imprinted DMR on the promotor of *TRIM61* had no heterozygous SNP inside the peak, which meant that it could not be detected by short-read ATAC-seq. The evidence supporting detection of the ASPs by scNanoATAC-seq also came from the random X chromosome inactivation. It is worth noting that ASPs on X chromosome were much more frequent than those on autosomes as expected in GM12878 (Fig. 1d), and they were mostly skewed toward the maternal alleles (Supplementary information, Fig. S6a). It was caused by the much higher percentage of the cells with the paternal X chromosome silenced in the GM12878 cell line, which was consistent with the previous discovery on DNase I hypersensitive sites of GM12878 by DNase-seq.^5^ These results showed the advantage of scNanoATAC-seq in identifying allele-specific chromatin accessibility.

As SVs may play an important role in cancer and other human genetic diseases, we took advantage of long reads generated by scNanoATAC-seq to detect large-scale SVs simultaneously. We evaluated the performance of SV detection in the K562 cell line using bulk ONT genome sequencing data as a benchmark. Precision and recall varied with the numbers of supporting cells (Fig. 1f; Supplementary information, Fig. S7a). Supported by at least five single cells sequenced, 7688 (64.6% of the benchmark) insertions and 6120 (67.7% of the benchmark) deletions were detected by our scNanoATAC-seq method in K562 cells, accounting for precision of 93.8% and 75.5%, respectively (Fig. 1g). For translocations (TRAs), we identified a classic TRA event of the *BCR-ABL1* fusion gene (Fig. 1h) known as the Philadelphia chromosome.^6^ We identified somatic SVs of K562 by filtering out germline SVs (Supplementary information, Data S1) and validated some of these somatic SVs by PCR (Fig. 1j, l; Supplementary information, Fig. S7a, b and Table S3). 65% of (17 out of 26) somatic insertions and 77% of (17 out of 22) somatic deletions were validated to be true. For example, we found a 322-bp somatic insertion in the intron of *DCLK3* (Fig. 1i, j). An 89-kb deletion event was also validated as a homozygous somatic deletion (Fig. 1l), which truncated *ZRANB1* and *CTBP2* simultaneously (Fig. 1k). Therein, CTBP2 was reported to inhibit leukemia proliferation^7^ which implied that we identified a potential loss-of-function SV of a tumor suppressor gene in K562 cells.

Next, we analyzed copy number variations (CNVs) in individual cells using scNanoATAC-seq data. Distinct CNV patterns were observed among different cell lines. eHAP1 is a fully haploid cell line without any large-scale CNVs^8^ and no large-scale CNVs were observed in our data (Supplementary information, Fig. S8a). GM12878 and HFF-1 were supposed to be diploid cell lines without obvious CNVs, while we detected subclones in the GM12878 cell line with large-scale CNVs on chromosomes 1 and 16 (Supplementary information, Fig. S8b). As HFF-1 is a male-derived cell line, we observed that the copy number of its chromosome X was half that of the autosomes as expected (Supplementary information, Fig. S8b). Large-scale CNVs were identified on chromosomes 7, 9, and X of K562^9^ and chromosome 13 of HEK293T^10^ (Supplementary information, Fig. S8c), consistent with previous works. Taken together, our method could accurately distinguish aneuploid cells from the normal diploid ones, and thus can be applied to the studies of cancer.

Finally, we exploited the long reads of scNanoATAC-seq to detect co-accessibility between pairs of neighboring peaks. The core assumption was that if two neighboring peaks were co-accessible in the same single cell, the length distribution of peak-supporting reads would be altered compared to the background (Supplementary information, Fig. S9a). We took the example of the co-accessible peak pair near the *SOX4* gene locus to demonstrate this strategy (Supplementary information, Fig. S9b, c). In this way, we identified 3868 pairs of co-accessible peaks from GM12878. Most of the pairs had a good correlation between the peak distance and the length of reads supporting them before we imposed the constraints on the peak distance (≤ 2-fold of the local median read length) (Supplementary information, Fig. S9d). We found more examples in GM12878 according to the global detection of co-accessibility. For instance, the promoter of *SERPINB8* was co-accessible with an enhancer annotated by ENCODE cCREs, which is 7 kb away from the promoter and located in the intron of *HMSD* annotated by ENCODE cCREs (Supplementary information, Fig. S9e). This provided direct evidence that these two regulatory elements were simultaneously accessible on the same allele in an individual cell. For another example, we can see that several enhancers and promoters on the histone gene cluster of chromosome 6 were co-accessible (Supplementary information, Fig. S9f).

In summary, scNanoATAC-seq is a TGS platform-based long-read single-cell ATAC sequencing method that can be applied in various biological fields. It can detect chromatin accessibility and genetic variants (including SVs, SNPs, and CNVs) within an individual cell simultaneously. Meanwhile, we revealed ASPs even without heterozygous SNPs inside a peak, which is not feasible for NGS platform-based short-read scATAC-seq. We provided the direct evidence of co-accessibility between neighboring peaks from scNanoATAC-seq, where the chromatin accessibility of two sites in the same single cell and in fact on the same allele was detected simultaneously by a long read. The cost of scNanoATAC-seq library is less than 2.5 dollars per cell under current sequencing depth. As the cost of ONT is still higher than that of NGS platform at the same sequencing depth, there is a trade-off between more fragments to enrich stronger epigenetic signals and longer reads to detect long read-specific features such as SVs.

## Supplementary information

Supplementary information, Figures and Data

Supplementary information, Table S1

Supplementary information, Table S2

Supplementary information, Table S3

## Data Availability

Data sequenced from human and mouse cell lines in this project were deposited in National Center for Biotechnology Information (NCBI). scNanoATAC-seq data (GSE194022)  and short-read bulk ATAC-seq data (GSE194023) of cell lines were deposited in Gene Expression Omnibus (GEO). The bulk genome of K562 sequenced on the ONT platform was deposited in Sequence Read Archive (SRA, PRJNA810364). Data related to human PBMCs were deposited in China National Center for Bioinformation (CNCB). The raw sequences of scNanoATAC-seq of human PBMCs were access controlled, PRJCA007999. The processed data of scNanoATAC-seq of human PBMCs was open access, OMIX001278. The publicly available 10× scATAC-seq data of human PBMCs were downloaded from the official website of 10× Genomics (https://www.10xgenomics.com/resources/datasets/1-k-peripheral-blood-mononuclear-cells-pbm-cs-from-a-healthy-donor-next-gem-v-1-1-1-1-standard-2-0-0). Data of 10× scATAC-seq of cell lines were from the work of Granja et al.^11^ and publicly available through GEO (GSE162690). The phased SNPs of GM12878 were provided by GIAB^3^ which is publicly available at https://ftp-trace.ncbi.nlm.nih.gov/giab/ftp/release/NA12878_HG001/NISTv3.3.2/GRCh38/.

## Code availability

All codes used in this paper are available on GitHub at https://github.com/Tanglab-Jiang/scNanoATAC-seq_paper.
