# Supplementary material for: scNanoATAC-seq: a long-read single-cell ATAC sequencing method to detect chromatin accessibility and genetic variants simultaneously within an individual cell
Source: Cell Res. 2022 Oct 11;33(1):83–6. doi: 10.1038/s41422-022-00730-x (PMC9810643; doi:10.1038/s41422-022-00730-x)
Supplement: Supplementary file 1 — Supplementary information, Figures and Data [file 41422_2022_730_MOESM1_ESM.pdf]

Supplementary information, Fig. S1

a

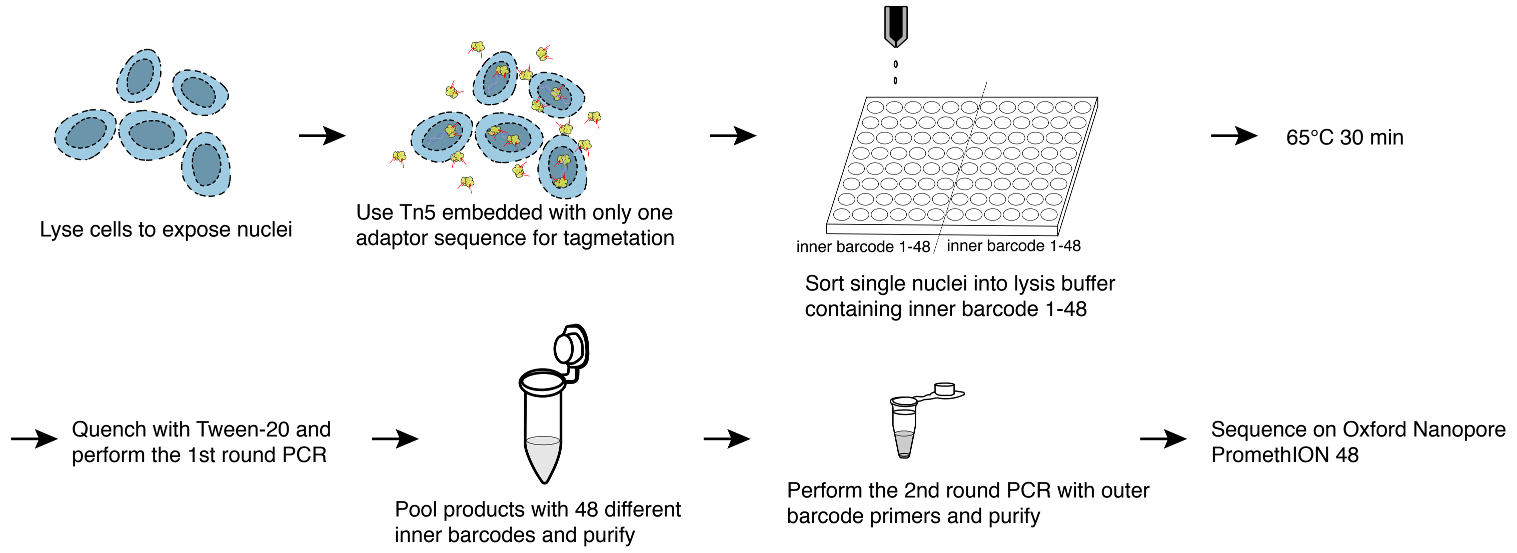

b

Library sequence

outer barcode / inner barcode

5'-ATCTCCTCATCTTGTGAAGTTGTTTCGGCTACACGACGCTCTTCCGATCTACTGGTGCAGCTTTGAACATCTAGTCGTCGGCAGCGTCAGATGTGTATAAGAGACAGNNNNNNNN...

TAGAGGAGTAGAACACTTCAACAAAGCCGATGTGCTGCGAGAAGGCTAGATGACCACGTCGAAACTTGTAGATCAGCAGCCGTCGCAGTCTACACATATTCTCTGTCNNNNNNNN...

...NNNNNCTGTCTCTTATACACATCTGACGCTGCCGACGACTAGATGTTCAAAGCTGCACCAGTAGATCGGAAGAGCGTCGTGTAGCCGAAACAACTTCACAAGATGAGGAGAT

...NNNNNGACAGAGAATATGTGTAGACTGCGACGGCTGCTGATCTACAAGTTTCGACGTGGTCATCTAGCCTTCTCGCAGCACATCGGCTTTGTTGAAGTGTTCTACTCCTCTA -5'

inner barcode / outer barcode

Supplementary information, Fig. S2

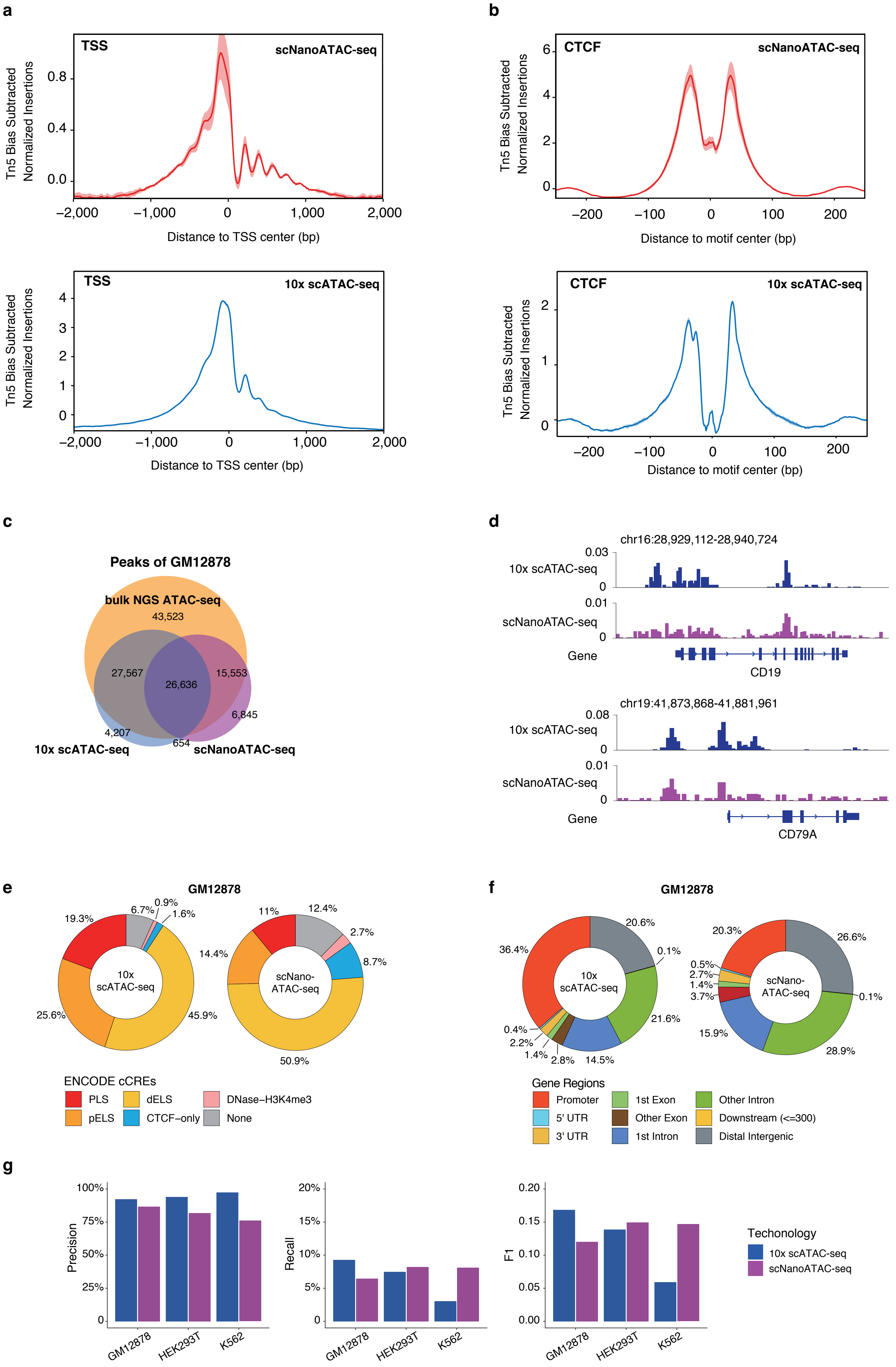

Supplementary information, Fig. S3

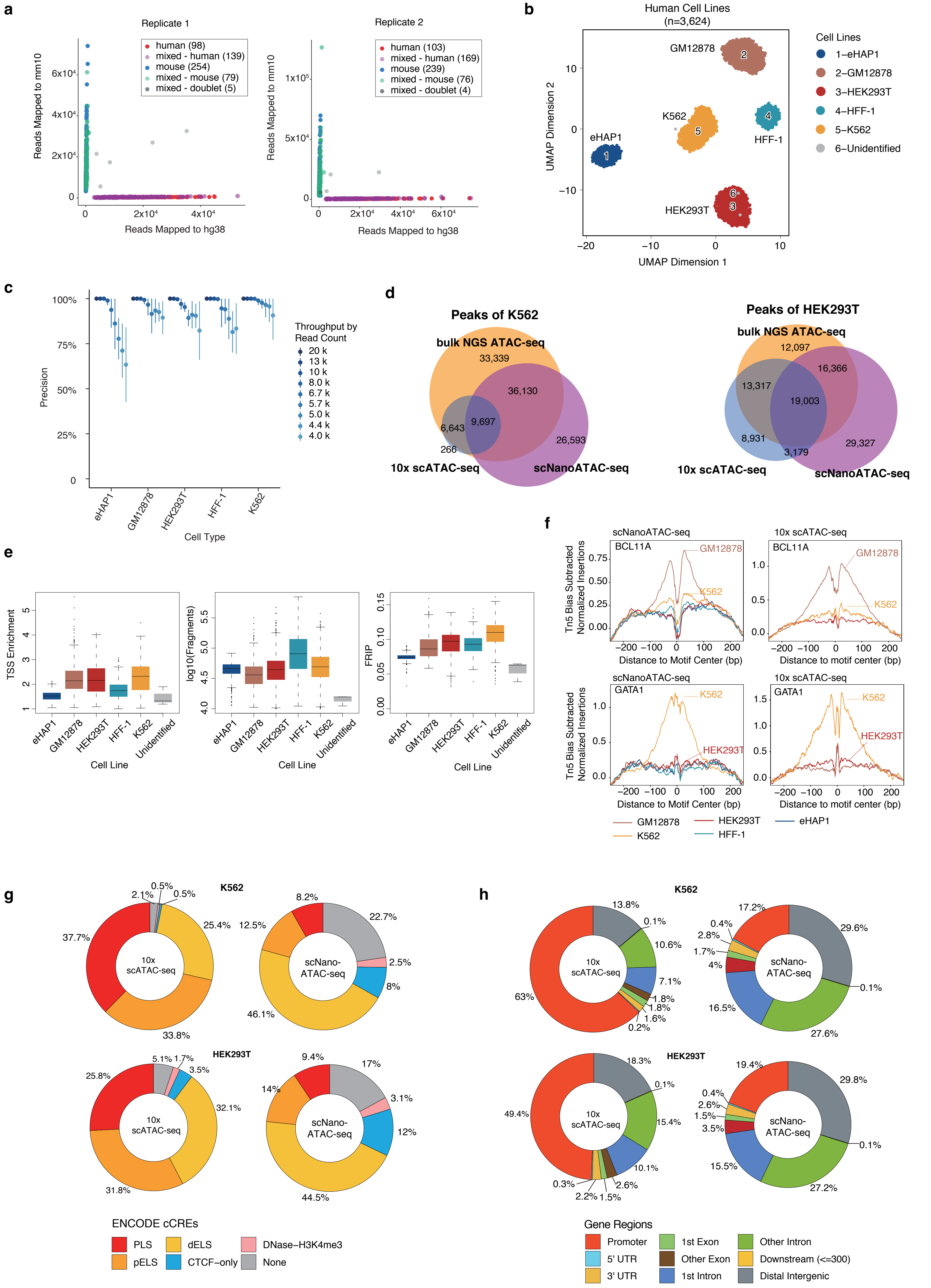

Supplementary information, Fig. S4

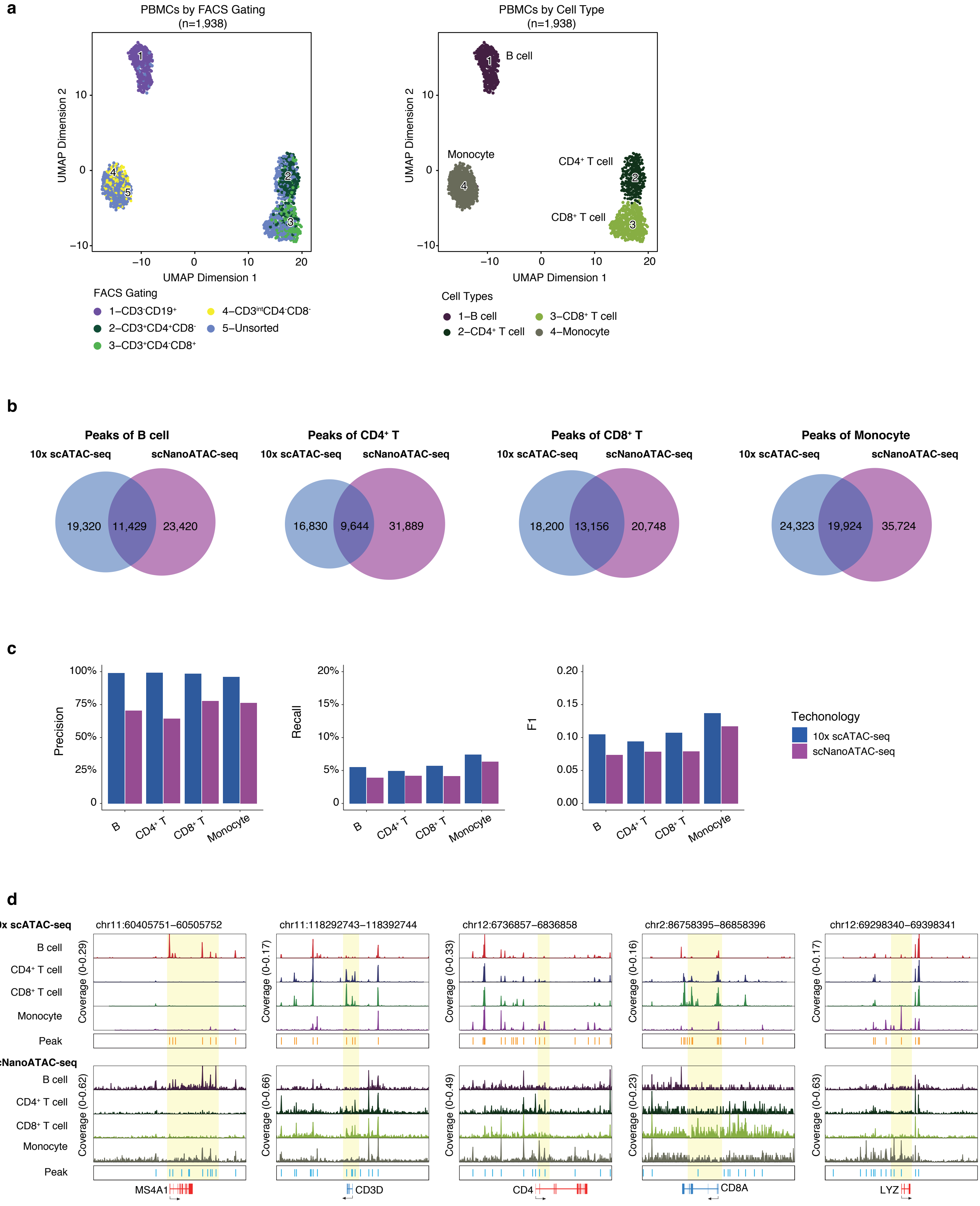

Supplementary information, Fig. S5

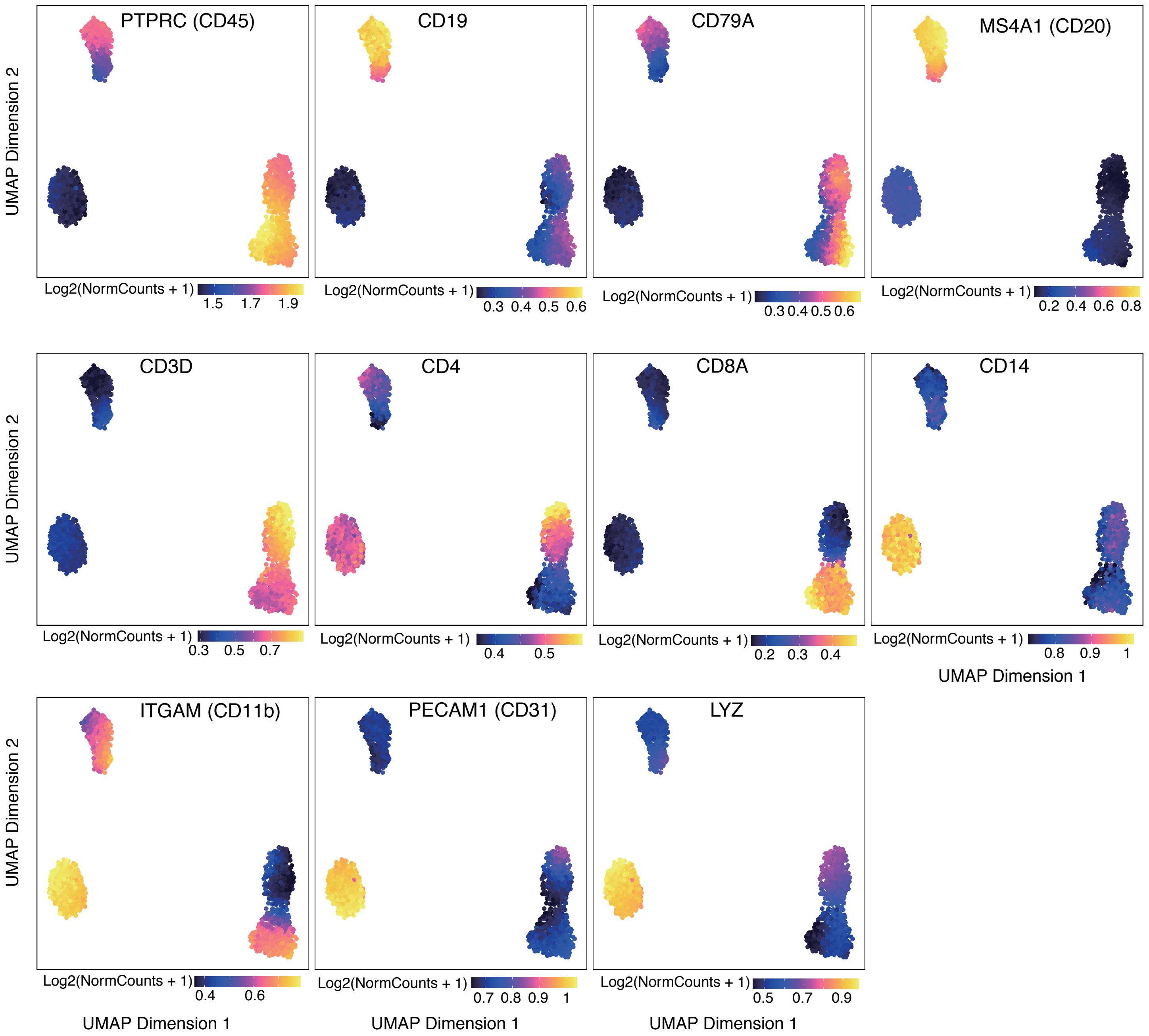

Supplementary information, Fig. S6

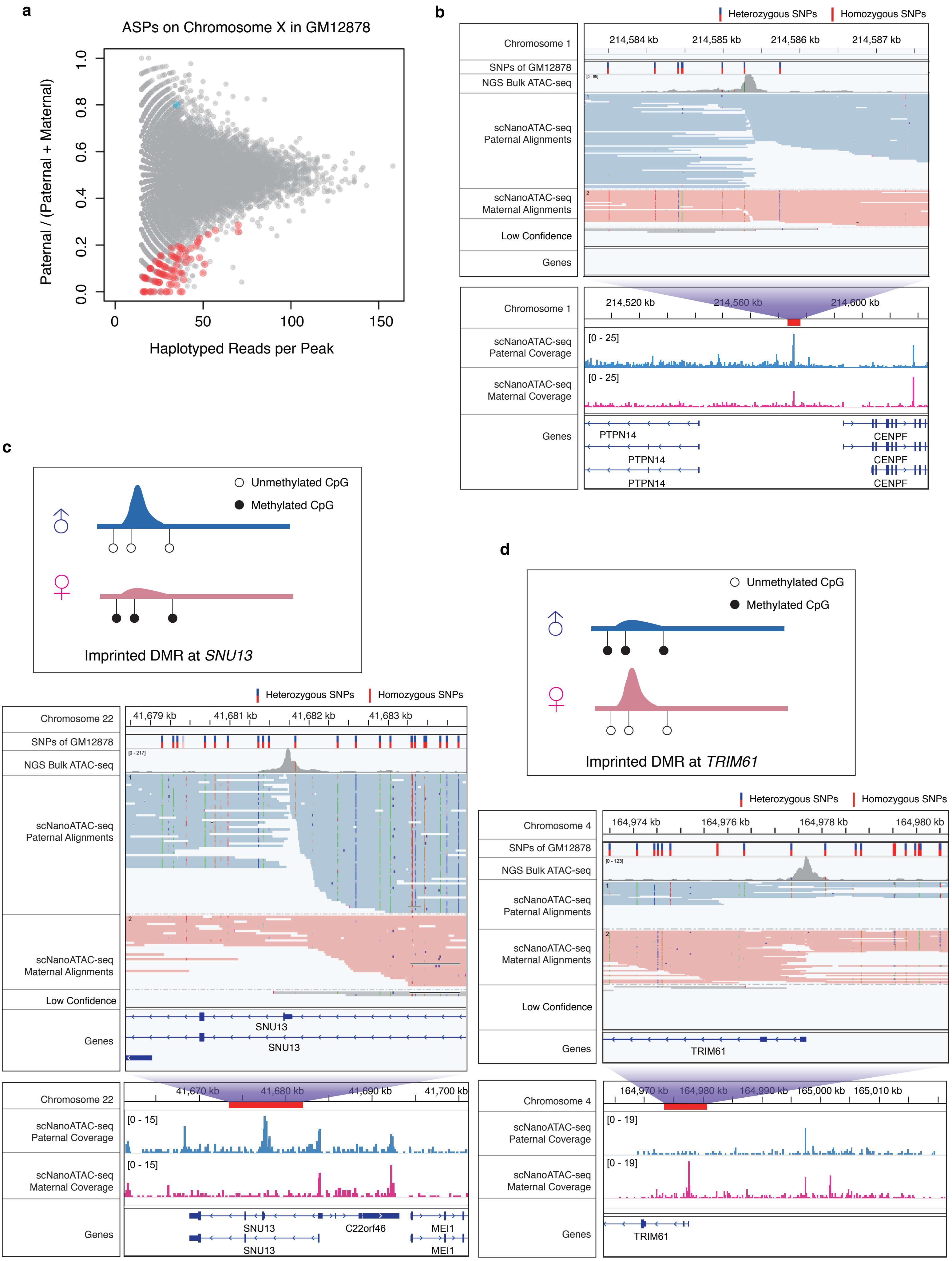

Supplementary information, Fig. S7

a

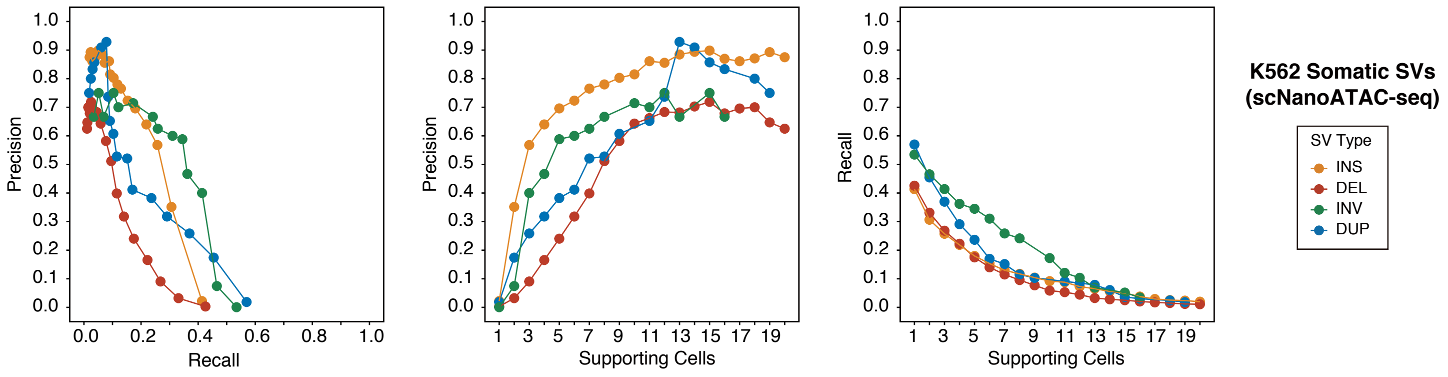

b

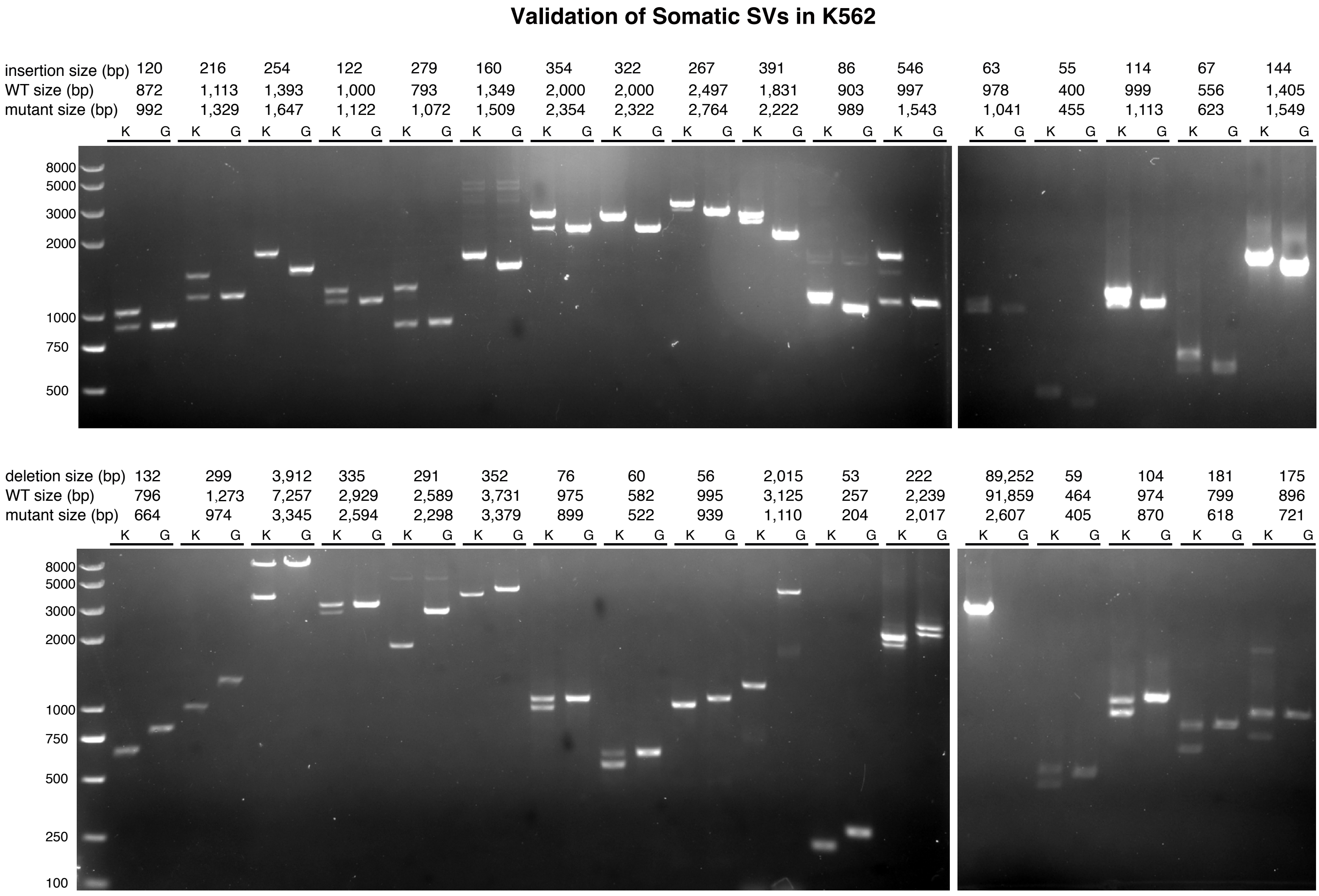

Supplementary information, Fig. S8

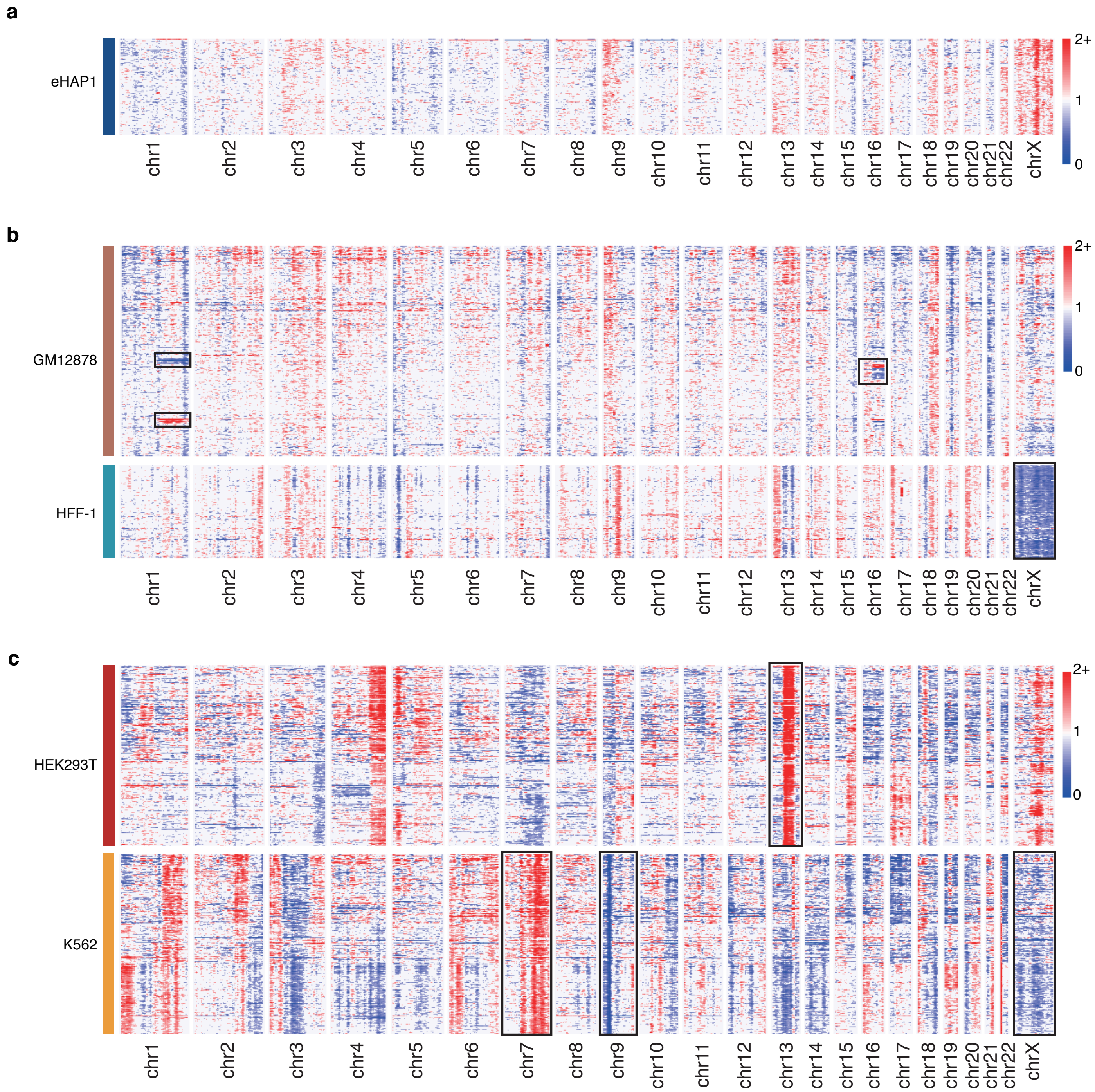

Supplementary information, Fig. S9

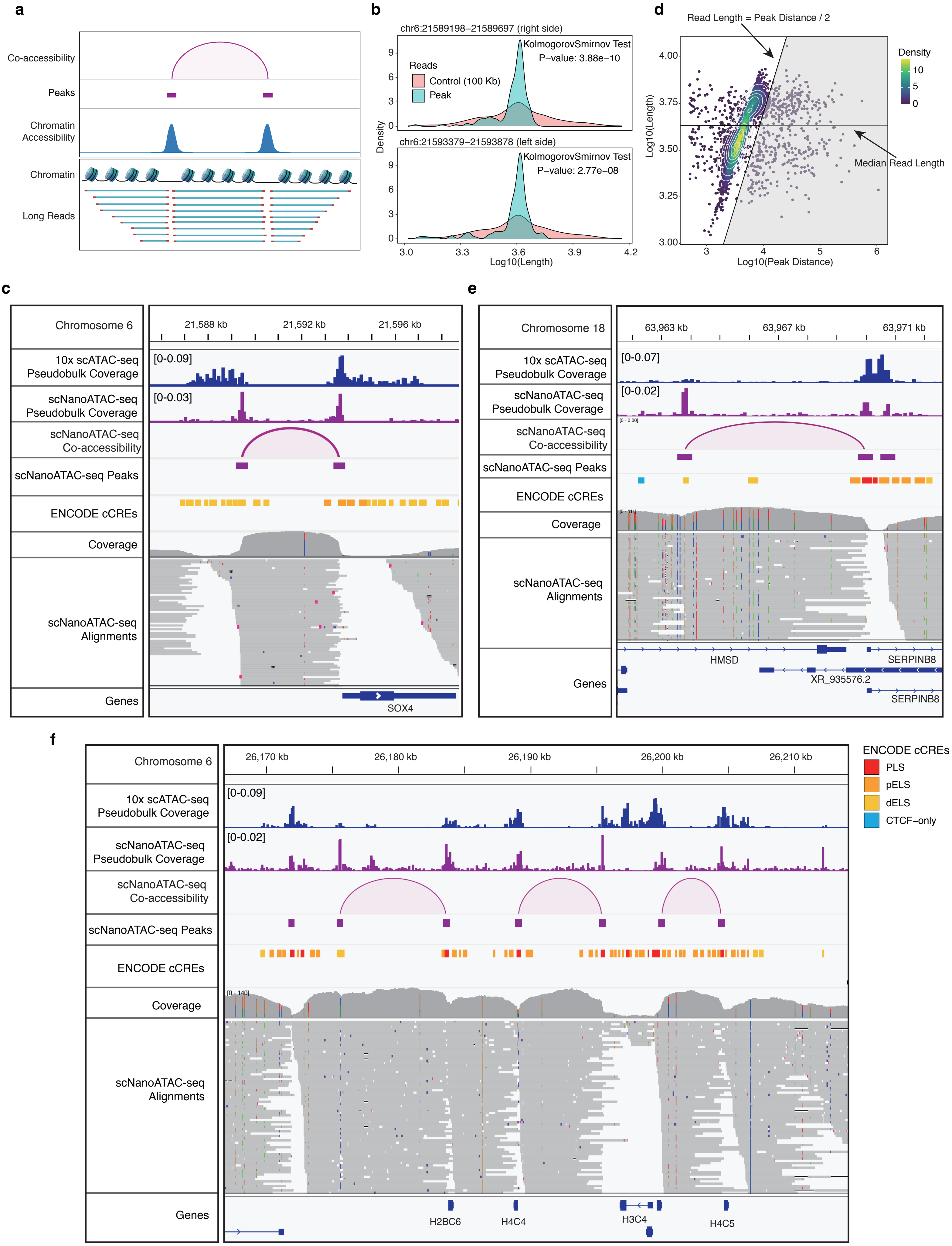

Supplementary information, Fig. S1 The workflow and library structure of scNanoATAC-seq. **a,** The schematic diagram for the overall workflow of scNanoATAC-seq. **b**, An example of the library structure of scNanoATAC-seq.

Supplementary information, Fig. S2 The performance of scNanoATAC-seq on the detection of chromatin accessibility in GM12878 cells. **a**, Signals of scNanoATAC-seq (top, red curves) and 10x scATAC-seq (bottom, blue curves) enriched around TSS. **b**, Signals of scNanoATAC-seq (top, red curves) and 10x scATAC-seq (bottom, blue curves) enriched around CTCF motifs. **c**, A Venn diagram showing the relationship among chromatin accessibility peaks of GM12878 called by bulk NGS ATAC-seq, 10x scATAC-seq and scNanoATAC-seq. **d**, Chromatin accessibility signals of GM12878 on *CD19* and *CD79A* were consistent between scATAC-seq of 10x and our method. **e**,**f**, Distribution of chromatin accessibility peaks on cCREs (**e**) and gene regions (**f**) identified by 10x scATAC-seq and scNanoATAC-seq in GM12878. **g**, Comparisons between the performance of 10x scATAC-seq and scNanoATAC-seq on the identification of cCREs in human cell lines.

Supplementary information, Fig. S3 The performance of scNanoATAC-seq on detection of chromatin accessibility in cell lines. **a**, Two replicates of experiments for testing cross-contamination of scNanoATAC-seq on the human-mouse mixing cell lines. **b**, The UMAP of five human cell lines based on scNanoATAC-seq. The labels of each cell line were identified by unsupervised clustering. Single cells with inferred identities inconsistent with the experiment design were labeled as unidentified. **c**, The effect of simulated read count per cell on the precision of unsupervised clustering of five human cell lines. Each condition of throughput was simulated for 15 times. **d**, Venn diagrams showing the relationship among chromatin accessibility peaks called by bulk NGS ATAC-seq, 10x scATAC-seq and scNanoATAC-seq in K562 cells (left) and HEK293T cells (right). **e**, Quality metrics of single cells by scNanoATAC-seq including TSS enrichment, fragment count and FRIP. **f**, GM12878 specific footprints of BCL11A and K562 specific footprints of GATA1 were consistent between 10x scATAC-seq and scNanoATAC-seq. **g**, **h**, Distribution of chromatin accessibility peaks on cCREs (**g**) and gene regions (**h**) identified by 10x scATAC-seq and scNanoATAC-seq in K562 and HEK293T cell lines.

Supplementary information, Fig. S4 The performance of scNanoATAC-seq on detection of chromatin accessibility in human PBMCs. **a**, The UMAP profiling human PBMCs sequenced by scNanoATAC-seq. Single cells were labeled according to fluorescence-activated cell sorting (FACS) gates (left) and cell types identified with marker genes (right). **b**, Venn diagrams showing the relationship between chromatin accessibility peaks called by 10x scATAC-seq and scNanoATAC-seq in four cell types of human PBMCs. **c**, Comparisons between the performance of 10x scATAC-seq and scNanoATAC-seq on the identification of cCREs in human PBMCs. **d**, Chromatin accessibility coverage tracks of marker genes for PBMCs detected by 10x scATAC-seq and scNanoATAC-seq respectively including *MS4A1(CD20)*, *CD3D*, *CD4*, *CD8A* and *LYZ.*

Supplementary information, Fig. S5 UMAP of PBMCs captured by scNanoATAC-seq. Single cells were colored by the marker gene activities calculated from chromatin accessibility signal on and around the gene bodies by ArchR.

Supplementary information, Fig. S6 ASPs identified by scNanoATAC-seq supported by well-studied epigenetic mechanisms in GM12878. a, ASPs on X chromosome in GM12878 were mainly biased toward the maternal alleles due to the random inactivation of X chromosome. **b**, An example for allele-specific chromatin accessibility in GM12878. Genotyped read ends representing imbalanced chromatin accessibility strength between alleles were sequenced by scNanoATAC-seq. This ASP was supported by short-read bulk ATAC-seq, which detected an allelic biased heterozygous SNP on a chromatin accessibility peak in GM12878. **c**, An ASP overlapped with an imprinted DMR on gene body of *SNU13*. As shown in the schematic diagram (top), the hypermethylation allele corresponds with less accessible chromatin. Haplotyped scNanoATAC-seq reads manifested allelic imbalanced chromatin accessibility according to the difference on the numbers of read ends inside the peak, which was supported by a biased heterozygous SNP towards the paternal allele in NGS bulk ATAC-seq (bottom). **d**, An ASP was on the promoter of *TRIM61*, which overlapped with a paternal imprinted DMR. The schematic diagram (top) showed the paternal -of-origin of imprinted methylation and allelic chromatin accessibility at *TRIM61*. Haplotyped scNanoATAC-seq reads manifested allelic imbalanced chromatin accessibility consistent with the allele of imprinting, while no heterozygous SNP inside the peak can be detected by short-read ATAC-seq in GM12878 (bottom).

Supplementary information, Fig. S7 *In silico* benchmarking of somatic SV identification and the PCR validation from the K562 cell line. a, The performance of somatic SV identification in K562 manifested by precision and recall varying with the numbers of supporting single cells. **b**, The PCR validation results of somatic insertions (top) and deletions (bottom) in K562 using GM12878 as the germline control. (K: K562, G: GM12878)

Supplementary information, Fig. S8 The single-cell CNV matrices called from scNanoATAC-seq data of five human cell lines. Values in the matrices are the fold changes of the copy number over the whole genome baseline of each cell line. We highlighted those single cells with their CNV regions in the heatmap. **a**, The CNV matrix of the fully haploid cell line eHAP1. **b**, The CNV matrices of diploid cell lines (GM12878 and HFF-1). **c**, The CNV matrices of aneuploid cell lines (HEK293T and K562).

Supplementary information, Fig. S9 Co-accessible peak pairs in the GM12878 cell line detected by scNanoATAC-seq. a, The co-accessibility was detected by altered read length distribution in the context of scNanoATACA-seq. **b,** We took the example of *SOX4* in GM12878 for co-accessible peak pair between the TSS and the upstream enhancer to compare the length distribution between the peak supporting reads and the 100 kb background around it. The distributions were significantly altered for the existence of another peak beside each of them. **c**, The reads supporting the co-accessible peak pair nearby *SOX4* were demonstrated in the genomic browser. The length of reads interlinking two peaks was constrained due to the co-accessibility. **d**, For all significant co-accessible pairs in GM12878, we showed the relationship between the median length of supporting reads and the distance between pairs of co-accessible peaks. In most cases, the median read lengths matched with peak distances. Those pairs with peak distances more than 2-fold the median read length were excluded. **e**, The promoter of *SERPINB8* was co-accessible with an enhancer (annotated by ENCODE) 7 kb far from it, which was located in the intron of *HMSD*. **f**, On

the histone gene cluster of chromosome 6, several enhancers and promoters are co-accessible.

Supplementary information

Materials and methods

General design of scNanoATAC-seq technique

In terms of experimental design, we adapted the plate-based scATAC-seq method of Chen et al.[1] for long-read sequencing from the following aspects (Supplementary information, Fig. S1a): (i) Embed Tn5 transposase with only one adaptor sequence instead of two adaptors to recover more original DNA fragments (Supplementary information, Fig. S1b). (ii) Isolate nuclei before tagmentation to improve transposition efficiency. (iii) Extend lysis time for more efficient Tn5 release. (iv) Lower denaturation and extension temperature to reduce DNA breakage and prolong extension time for DNA synthesis to ensure sufficient amplification of long DNA fragments. Each scNanoATAC-seq library contained 480 or 960 single cells and was sequenced using one PromethION flow cell on Oxford Nanopore Technology (ONT) platform.

In terms of bioinformatics, we adapted analysis pipelines to accommodate the long reads of scNanoATAC-seq data, including (i) Unlike taking the whole reads as chromatin accessibility signal in short-read scATAC-seq analysis, we extracted read ends of scNanoATAC-seq for signal analysis (Fig. 1a). We manifested that chromatin accessibility signal retained by long-read scATAC-seq is similar to that produced by short-read scATAC-seq on the human cell lines studied. (ii) As the fragment size increased from less than 300 bp (NGS) to several kilobases (TGS), the number of heterozygous single nucleotide polymorphisms (SNPs) detected per fragment increases more than ten folds. Therefore, we analyzed allele-specific chromatin accessibility using heterozygous SNPs around the peak rather than inside the peak, which was not achieved by short-read ATAC-seq[2,3]. (iii) As long-read sequencing is powerful on the detection of large-scale SVs[4], we designed a SV calling pipeline for scNanoATAC-seq data and identified somatic SVs in the K562 cell line, some of which were validated by polymerase chain reaction (PCR). (iv) Co-accessible neighboring peaks were detected by scNanoATAC-seq, which provided direct evidence of co-accessibility from the same individual cell.

Cell culture

K562 cells were cultured in RPMI-1640 medium (Gibco; 11875093) supplemented with 10% fetal bovine serum (Gibco; 26140079). HEK293T cells, HFF-1 cells and MEF cells were cultured in Dulbecco's modified eagles's medium (DMEM,Gibco; 11995040) supplemented with 10% fetal bovine serum. GM12878 cells were cultured in RPMI-1640 medium supplemented with 15% fetal bovine serum. eHAP1 cells were cultured in IMDM (Gibco; 12440053) supplemented with 10% fetal bovine serum. The K562, HEK293T and HFF-1 cell lines were authenticated by STR DNA profiling. The GM12878 and eHAP1 cell lines were authenticated by SNP genotyping based on whole genome sequencing. Mouse embryonic stem cells (mESCs) were cultured without feeders in equal volume of DMEM (DMEM/F-12, Gibco; 11320033) and Neurobasal Medium (Gibco; 21103049) supplemented with 1,000 U/ml leukemia inhibitory factor (LIF, Millipore, ESG1107), 0.5× N-2 (Gibco, 17502048), 0.5× B-27 (Gibco, 10889038), 50 μg/ml Bovine Serum Albumin (BSA,

Sigma-Aldrich, A3311), 2 mM L-Glutamine (Gibco, 2503081), 0.1 mM β-Mercaptoethanol (Sigma-Aldrich, M7522), 1× MEM Non-Essential Amino Acids Solution (NEAA, Gibco, 11140050), 100 U/ml Penicillin and 0.1mg/ml Streptomycin (Gibco, 15140122).

Adherent cell lines (HEK293T, HFF-1, eHAP1, MEF, mESC) were digested with 0.25% Trypsin-EDTA (Gibco; 25200056) while suspension cell lines (K562, GM12878) were harvested directly to prepare single cell suspensions. Cells were then pelleted and resuspended with QuickFreezing-M cell freezing medium (Biodragon; KX0310041). The frozen cells can be stored in liquid nitrogen for months before scNanoATAC-seq.

The isolation and FACS sorting of PBMCs

The study was approved by the Ethics Committee of Peking University Third Hospital (License No. IRB00006761-M2016170), and the blood was sampled from a colorectal cancer patient with informed consents.

The PBMCs were isolated with Histopaque-1077 (Sigma_Aldrich; 10771) according to the product instructions. Briefly, 3 mL of whole blood was gently layered onto the 3 mL of Histopaque-1077 equilibrated to room temperature in a 15-mL conical centrifuge tube. The tube was then centrifuged at 400 × g for 30 minutes at room temperature. After centrifugation, the opaque interface containing mononuclear cells was carefully transferred into a new tube. Cells were washed twice with DPBS (Sigma_Aldrich; D8537) solution and resuspended with FACS buffer (DPBS supplemented with 0.5% Bovine serum albumin (Milenyi Biotec; 130-091-376)).

Cell suspension was stained with the following antibodies: APC-Cy7 anti-human CD45 (Biolegend; 368516), FITC anti-human CD3 (Biolegend; 300306), APC anti-human CD19 (Biolegend; 392504), BV421 anti-human CD8 (Biolegend; 344748), BV510 anti-human CD4 (Biolegend; 344634). B cells (7-AAD$^-$CD45$^+$CD19$^+$CD3$^-$), CD4$^+$ T cells (7-AAD$^-$CD45$^+$CD19$^-$CD3$^+$CD4$^+$CD8$^-$), CD8$^+$ T cells (7-AAD$^-$CD45$^+$CD19$^-$CD3$^+$CD4$^-$CD8$^+$), monocytes (7-AAD$^-$CD45$^+$CD19$^-$CD3^{int}CD4$^-$CD8$^-$) were sorted into 1.5-mL centrifuge tube (Axygen; MCT-150-C-S) coated by FACS buffer. The collected cells were stored in QuickFreezing-M cell freezing medium until scNanoATAC-seq.

Library construction of scNanoATAC-seq

Cells (100,000-200,000 cells before freezing) were quickly thawed at 37°C and centrifuged at 500 × g for 5 min at 4°C. Then cells were resuspended with Omni-ATAC lysis buffer (10 mM Tris-HCl (pH 7.4), 10 mM NaCl, 3 mM $MgCl_2$, 0.1% IGEPAL CA630, 0.1% Tween-20, 0.01% digitonin) and incubated on ice for 3-5 min.

Immediately after lysis, nuclei were centrifuged at 800 × g for 5 min at 4°C and resuspended with 50 μL of tagmentation mix (33 mM Tris-acetate (pH 7.8), 66 mM Potassium acetate, 10 mM Magnesium acetate, 16% Dimethylformamide, 0.01% digitonin and 10 μL of 8.3 μM Tn5 transposase). The customized transposase we used with only one adaptor sequence (TCGTCGGCAGCGTCAGATGTGTATAAGAGACAG) was purchased from Novoprotein. The reaction was incubated on a thermomixer at 37°C, 800 rpm, for 30 min and stopped by 50 μL of tagmentation stop buffer (10 mM Tris-HCl (pH 8.0), 20 mM EDTA).

After that, nuclei were stained with DAPI. Single nucleus was flow sorted into each well of 96-well plate containing 4 μL of lysis buffer (50 mM Tris-HCl (pH 8.0), 50 mM NaCl, 20 μg/mL Proteinase K, 0.4% SDS, 5 μM Inner Primer (CTACACGACGCTCTTCCGATCT-[24 bp inner barcode 1-48]-TCGTCGGCAGCGTCAGAT)) and then incubated at 65 °C for 30 min to deplete nucleosome and disassociate the transposase enzyme.

SDS was then quenched with 4 μL of 10% Tween-20, and PCR was performed immediately by the addition of 4 μL of 5X PrimeSTAR GXL Buffer, 1.6 μL of dNTP Mixture (2.5 mM each), 0.4 μL of PrimeSTAR GXL DNA Polymerase (TaKaRa; R050) and 6 μL of H_2O. The 96-well plate was then vortexed and thermal cycled as follows: 68°C for 10 min; 95°C for 1 min; 16 cycles of 94°C for 15 s, 63°C for 30 s, 68°C for 8 min and finally 68 °C for 5 min.

5 μL of each of 48 wells with different inner barcodes was then pooled and purified with 1X AMPure XP beads (Beckman Coulter; A63882) and eluted into 25 μL of H_2O. The second amplification was carried out in a 50 μL GXL DNA Polymerase system with 1 μM Outer Primer (ATCT-[24 bp outer barcode 73-96]-CTACACGACGCTCTTCCGATCT). The incubation program was as follows: 95°C for 1 min, then 4 cycles of 94°C for 15 s, 63°C for 30 s, 68°C for 8 min and finally 68 °C for 5 min. The amplified products were then purified with 1X AMPure XP beads twice.

20 library products with different outer barcodes (960 cells in total) were pooled together and sequenced on one Oxford Nanopore PromethION 48 (Oxford Nanopore Technologies; R9.4.1) by Grandomics Biosciences (Beijing, China).

Bulk NGS platform-based ATAC-seq

Nuclei were isolated with Omni-ATAC lysis buffer. Then nuclei were resuspended in 50 μL of tagmentation mix with Tn5 from the NovoNGS DNA Library FlashPrep Kit for Illumina (Novoprotein, N233). The tagmentation reaction was performed on a thermomixer at 800 rpm, 37 °C, for 30 min. To stop the tagmentation reaction, 50 μL of tagmentation stop buffer (10 mM Tris-HCl (pH 8.0), 20 mM EDTA (pH 8.0)) was added to the tagged nuclei. Then nuclei stained with DAPI were sorted into 200-μl tubes by FACS. The nuclei were resuspended in lysis buffer (50 mM Tris-HCl (pH 8.0), 50 mM NaCl, 20 μg/mL Proteinase K, 0.4% SDS) and incubated at 65°C for 30 min to release Tn5. Equal volume of 10% Tween-20 was added to ~30 ng lysed DNA product. 2X KAPA HiFi HotStart Ready Mix (KAPA, KK2602) and S5xx/N7xx Nextera Index Primer Mix were added to the tube. Then amplification reaction was performed as follows: 72°C 10 min; 98°C 5 min; 15 cycles of 98°C 10 s, 63°C 30 s and 72°C 20 s; 4°C hold. Libraries were size selected (200-700 bp) with 2% agarose gel and sequenced on Illumina NovaSeq 6000.

Bulk DNA extraction and validation of structure variations

Genomic DNA (gDNA) of K562, GM12878 and eHAP1 cells was extracted using the QIAGEN DNeasy Blood and Tissue Kit (QIAGEN, 69504) following the manual's instructions.

The somatic insertions and deletions occurred within genes in K562 called from scNanoATAC-seq were validated by PCR. PCR primers were designed to ensure that the products span the breakpoints.

The PCR products were run on 1% agarose gels.

Bulk whole genome sequencing library construction for Nanopore sequencing platform

The gDNA of K562 cells was submitted to the Novogene Company for ONT library construction and sequencing on Oxford Nanopore PromethION 48 (Oxford Nanopore Technologies; R9.4.1). The mean length of reads is around 10 kb.

Basic processing of data produced by scNanoATAC-seq

1) The raw data produced by ONT sequencing was recorded in electric signal. To convert electric signal files into fastq format, we used Guppy[5] (v4.0.11) to implement base calling.
2) To demultiplex single cells from a long-read nanopore library, we used nanoplexer (v0.1) (https://github.com/hanyue36/nanoplexer) to identify single-cell barcodes in noisy long reads. According to our library structure of dual single-cell barcodes, demultiplexing was performed twice on outer and inner barcodes in succession.
3) Adaptors of demultiplexed reads were removed by cutadapt[6] (v3.2). The adaptor sequences trimmed include 'TCGTCGGCAGCGTCAGATGTGTATAAGAGACAG' at 5' ends and 'CTGTCTCTTATACACATCTCCGAGCCCACGAGA' at 3' ends. If at least 12 bases are matched with adaptor sequences in a read, those matched bases will be removed. As sequencing errors happen frequently for long-read ONT sequencing, the tolerance of the proportion of mismatched bases was set to 20%. We also filtered out reads shorter than 1 kb to preserve long reads only.
4) Trimmed reads were aligned to the reference genome hg38 or mm10 by minimap2[7] (v2.17-r941). We aligned reads with `-ax map-ont` to adapt the program to nanopore sequencing and with `--MD` to add the MD tags into bam files for SV calling later. Reads with mapping quality less than 30 were filtered out by samtools[8] (v1.14). PCR duplicates were removed by single cell with `samtools rmdup` command.

Extraction of chromatin accessibility signal from scNanoATAC-seq reads

The chromatin accessibility signal is captured by transposition events of Tn5 enzyme molecules. As a consequence, the chromatin accessibility signal is represented by the ends of long reads by scNanoATAC-seq. In practice, mapped long reads were converted from bam to bed format by `bamtobed` command of bedtools[9] (v2.30.0). Then the coordinates of both ends of the reads were extracted by `bedtools flank` command with a 1 bp flanking size. The generated bed files recorded the chromatin accessibility signal in each single cell and were merged into fragment files. The fragment file of scNanoATAC-seq is equivalent to that of canonical short-read scATAC-seq both biologically and computationally, except that the information about fragment size is eliminated here.

Analysis of single-cell chromatin accessibility by ArchR

To make our analysis of the scNanoATAC-seq signal easy and reproducible, we performed most of the basic analysis by ArchR[10] (v1.0.2), which is a versatile R[11] (v4.1.0) implementation for single-cell ATAC-seq analysis. The fragment files of libraries were input to `createArrowFiles` function of ArchR to convert chromatin accessibility coordinates into arrow files, which store data in HDF5 format for efficient reading and writing. We disabled quality control in this step by setting `maxFrags=1e6` and `minTSS=1`. The fragments located on chromosome Y and mitochondrial

DNA were filtered out by default.

After the generation of all the arrow files, we loaded them into ArchR and combined them into ArchR projects. Next, we did single cell quality control, which kept single cells with more than 10,000 fragments. Tile matrices stored in arrow files divided by 500-bp bins were loaded and reduced by `addIterativeLSI` function. This function conducted a logarithmic term frequency–inverse document frequency (log TF-IDF) transformation by two iterations and singular value decomposition (SVD) to reduce the tile matrix to an SVD matrix spanned by the top 30 principal components. The UMAP (uniform manifold approximation and projection for dimension reduction) was computed from the SVD matrix with `minDist=1` to control graph density. Single cells were clustered by `addClusters` function using Seurat methods. `maxClusters` was set to control the number of clusters on demand of making unsupervised clustering consistent with the prior knowledge of cell types in each project.

To generate pseudo-bulk coverage of scNanoATAC-seq by cell line, `addGroupCoverages` function was called to group single cells by cell line and divide them into pseudo bulks to merge. Coverage tracks in bigwig format were generated by `getGroupBW` function with `maxCells=1e4` that abolished the limitation on the number of cells to be merged and `normMethod='nFrags'` that normalized coverage by the fragment number of pseudo bulks. Meanwhile, peak calling was performed by `addReproduciblePeakSet` function. This function implemented peak calling by MACS2[12] and further controlled false discovery by examination of reproducibility. We set `additionalParams` as `--nomodel --llocal 500000`, which means peaks were detected locally against the baseline calculated from each 500 kb window rather than against the global baseline. Global detection significantly increased false discovery of peaks on regions with gains of copy number. We also set the cut-off of q-value to 0.01 to control false discovery. Finally, we got peak sets by cell line in GRange objects and output them in bed format.

Once peak sets were called for cell lines sequenced by scNanoATAC-seq, we did motif enrichment analysis on this basis. We ran `addPeakMatrix` and `addBgdPeaks` functions to quantify signal strength in peak and background regions. Then those peaks were annotated by `addMotifAnnotations` function with cisbp[13] database of motifs. The final visualization of motif enrichment of cell lines was implemented by `plotFootprints` function, setting `smoothWindow=10` to smooth enrichment curves.

Basic processing of short-read bulk ATAC-seq data

Sequencing adaptors of raw Illumina paired-end reads were removed by cutadapt, including 'AGATGTGTATAAGAGACAG' at 5' ends and 'CTGTCTCTTATACACATCT' at 3' ends. It also filtered reads with sequencing quality scores < 20 or size < 30 bp at either end. Trimmed paired-end reads were aligned to the reference genome (hg38 or mm10) by Bowtie2[14] (v2.4.2) with `--no-unal` parameter to abandon unaligned reads. Reads with mapping quality less than 30 were filtered out by samtools. Duplicated reads were removed by picard MarkDuplicates`, including both PCR duplicates and optical duplicates of Illumina sequencing.

Short-read ATAC-seq peaks were called from short ATAC-seq reads by MACS2[12] (v2.2.7.1). We

ran `macs2 callpeak` command on bam files with `--nomodel --shift -75 --extsize 150` for short-read ATAC-seq and kept the other parameters default. The false discovery rate of peak calling was controlled to 0.01 by setting `-q 0.01`.

Evaluation of peak calling from scNanoATAC-seq

To evaluate the performance of peak calling, we compared the peaks called from scNanoATAC-seq data with both ENCODE cCREs[15] and short-read ATAC-seq peaks as benchmarks. Computationally, if there is any base overlap between two peaks, we defined them as equivalent. Overlaps were found by `findOverlaps` function in the GenomicRanges[16] (v1.44.0) R implementation.

To make a straightforward evaluation, we compared the peaks of scNanoATAC-seq with short-read ATAC-seq called from the same sample type. For short-read bulk ATAC-seq, we called peaks by MACS2 from bam files directly and used them as benchmark sets. For short-read scATAC-seq, we down-sampled single cells of the benchmark dataset to the same number of cells produced by scNanoATAC-seq and called benchmark peak sets by ArchR. The benchmark 10x scATAC-seq dataset for human cell lines was obtained from the work of Granja et al.[10], including GM12878, K562, and HEK293T. The benchmark scATAC-seq dataset of human PBMCs was obtained from the website of 10x Genomics. We visualized overlaps between peak sets in the Venn diagram plotted by the eulerr[17] (v6.1.1) R implementation.

We also made an evaluation of peak calling based on ENCODE cCREs[15], which is a dataset of cis-regulatory elements for both human and mouse. As including 190 tissues, 168 cell lines, 87 primary cells and 45 in vitro differentiated cells of human, ENCODE cCREs is a fully recalled dataset of cis-regulatory elements. It classified elements into five types: promoter-like signature (PLS), proximal enhancer-like signature (pELS), distal enhancer-like signature (dELS), DNase-H3K4me3 and CTCF-only. In this section, we intersected tested peaks with cCREs and used precision and recall as evaluation metrics. Precision is defined by TP/(TP+FP) and recall is defined by TP/(TP+FN), where true positive (TP) is the intersection of the test and the benchmark set, false positive (FP) is the complement of the benchmark in the test set and false negative (FN) is the complement of the test set in the benchmark set. We also used the F1 score as a comprehensive metric for evaluation of peak calling, which is the harmonic mean of precision and recall defined by 2/(1/precision+1/recall).

Evaluation of cross-contaminations of scNanoATAC-seq technique

To evaluate cross-contamination among single cells introduced by constructing scNanoATAC-seq libraries, four cell lines (two human and two mouse cell lines) were equally mixed before library construction. The experiment was performed with two replicates. Data was processed by the following steps:

1) To map human-mouse mixed reads to the reference genome, we merged hg38 and mm10 to build a mixed reference genome. It was indexed by minimap2 with a crucial parameter `-I 10G`, which made minimap2 read all the bases of the mixed genome into the minimizer to ensure proper alignment. As reads were generated by the ONT platform, `-x map-ont` was also set for indexing.
2) After indexing the mixed genome, cross-contamination libraries by scNanoATAC-seq were

processed by the same basic procedure as mentioned above, except that reads were mapped to the mixed reference genome rather than a single species genome.

3) Once mixed reads were aligned and filtered, we counted the read numbers for mm10 and hg38 alignments, respectively, for each single cell. Then we selected high-quality single cells for evaluation of cross-contamination. Single cells with fragment numbers < 3000 were filtered out, because the origin of low-quality cells was confusing, which is a criterion different from the previous one for scATAC analysis.
4) The origin of single cells was determined by the species of reference genome to which a single cell was majorly mapped. If the proportion of reads mapped to the major genome in a single cell was less than 0.9, this cell would be labeled as cross-contaminated or doublets. Otherwise, a cell would be regarded as not contaminated.

Estimation of the optimal throughput of single cells for scNanoATAC-seq

To estimate the optimal throughput based on sequencing data we've collected beyond, we simulated single-cell data by sampling reads from pseudo bulks of each cell line without replacement to specific numbers of reads per cell. Based on prior knowledge of scNanoATAC-seq libraries, the read number of each ONT sequencing run was about 20 million. As the yield of reads per run is relatively stable, the sequencing depth per cell is determined by the throughput of cells. Therefore, the fragment number (counted by read end) of each simulated single cell is 2 * 20 M / throughput. We simulated 500 single cells for each cell line and repeated the simulation for 15 times for each throughput condition.

Once we got the simulated datasets, we evaluated the performance of scNanoATAC-seq under specific throughputs by the precision of unsupervised clustering to distinguish 5 cell types. As the fragment number per cell was set to be uniform across single cells in simulated data, the process of `addIterativeLSI` that removes principal components correlated with fragment number was no longer suitable. We turned off this function by adding a uniform distributed dummy variable as `depthCol` to be regressed in ` addIterativeLSI ` and set `corCutOff=1` to disable the filtering of principal components. Next, we clustered simulated cells by `addClusters` with `maxClusters=5`. If single cells failed to be clustered into five clusters, that simulation dataset will be eliminated. Precision of clustering was defined by the ratio of the number of correctly clustered cells against the number of total cells of each cell line. A single cell was defined as correctly clustered if it was in the cluster which that cell line dominated. Precision values were calculated by cell type and throughput condition and finally illustrated in a boxplot.

Identification and validation of ASPs

For calling allele-specific chromatin accessibility, both short-read ATAC-seq and long-read scNanoATAC-seq exploit heterozygous SNPs on reads. As most of the GM12878 genome was already phased by GIAB[18,19], providing a benchmark SNP calling set, we used this diploid cell line as an object of study. For both long-read and short-read ATAC-seq, allele-specific events were defined by significantly biased allele frequency on heterozygous SNPs.

For scNanoATAC-seq data, we genotyped each long read with multiple known heterozygous SNPs. As heterozygous SNPs of GM12878 have already been phased by GIAB[18,19], we exploited it to

distinguish paternal and maternal reads by the tagging function of whatshap[20] (v1.2.1). The command for tagging was `whatshap haplotag -ignore-read-groups -o $output_bam -reference $hg38_fasta -regions $chr $ref_snp $input_bam`, where `-regions $chr` was used to parallelize the computation by chromosome. The genotyped long reads in the bam files were then converted to bed files. The 1-bp flanking regions of each fragment were extracted as previously mentioned. So far, we've got chromatin accessibility signal by haplotype for identification of ASPs.

Since phased SNP sets are not common for most of samples studied, we built a pipeline to phase known heterozygous SNPs with scNanoATAC reads and used the de novo phased SNP set to genotype our reads. The known heterozygous SNPs of GM12878 was also provided by GIAB. We phased them using HapCUT2[21](v1.3.1) with ONT mode and error analysis mode on, and genotyped reads using `haplotag` function of whatshap that mentioned above. To evaluate the accuracy of phasing with scNanoATAC reads, we conducted switch error analysis using `trio-switch-rate` command of bcftools[8] (v1.14). Besides the phased SNPs of GM12878 to be evaluated, pedigree information of GM12878 was also input, including SNPs of NA12891 (paternal) and NA12892 (maternal) provided by the 1000 Genomes Project[22].

For short-read ATAC-seq data, we analyzed aligned reads to call allele frequency on known heterozygous SNPs. Bam files were input into freeBayes[23] (v1.3.5) with the SNP benchmark of GM12878 from GIAB[18,19] in vcf format. Parameters were set to `--min-alternate-fraction 0 --min-alternate-count 0 --min-alternate-total 0 --only-use-input-alleles -@ $ref_snp`, where `min-alternate` disabled the filter of the minimum count of minor alleles and `-@ $ref_snp` assigned the reference SNPs used to call allele frequency. To call SNPs efficiently, we parallelized the computation by chromosome and finally concatenated those vcf files by `concat` command of bcftools.

In the stage of identification, we called ASPs from long-read scNanoATAC-seq data. We used the previously mentioned GM12878 peaks detected by scNanoATAC-seq as candidates for calling ASPs. Peaks intersecting with less than 15 genotyped long read ends were filtered out, because allelic bias is unlikely to be statistically significant under this sample size. We counted paternal and maternal ends respectively and tested statistical significance of the bias of allele frequencies on each candidate peak with binominal test. The null hypothesis is that the chromatin accessibility of the maternal or paternal chromosome on one specific peak is equal. This hypothesis test was expressed in `binom.test(c(mat, pat), p=0.5)` by the stats[11] (v4.1.0) package, where `mat` and `pat` are counts of allele-specific ends inside each peak. After testing all candidate peaks, we made multiple test corrections on the P value with methods of Benjamini-Hochberg[24] by `p.adjust(p, method='fdr)` function. Setting FDR to 0.05, significantly biased peaks were divided into paternal or maternal specific peaks according to the strength of signal by haplotype.

In the stage of validation, we exploited short-read bulk ATAC-seq as the benchmark to evaluate the reliability of ASPs called by scNanoATAC-seq. As allelic bias is impossible to be measured by short-read ATAC-seq without heterozygous SNPs inside a peak, we gave a definition to the verifiable ASP of long-read ATAC-seq. It is an ASP intersecting with at least one heterozygous SNP, and the locus is covered by more than 15 reads of short-read ATAC-seq. We made binominal tests

from the allele frequencies on heterozygous SNPs by short-read ATAC-seq for verifiable biased peaks of scNanoATAC-seq only. The null hypothesis and multiple test correction were the same as those in the stage of identification. Once any SNP inside an ASP is significantly biased, we will examine the consistency of direction of allelic bias between long-read and short-read ATAC-seq. If statistical significance and consistency of bias direction are both validated by short-read ATAC-seq, the ASP detected by scNanoATAC-seq is verified. In the end, the precision of ASP identification was obtained by dividing the number of verified ASPs by the number of verifiable ASPs.

Calling SVs from scNanoATAC-seq datasets

We performed SV identification for each single cell from scNanoATAC-seq libraries with cuteSV[25] (v1.0.10), an SV caller suitable for nanopore long reads. Single-cell bam files were input to cuteSV. We set the recommended parameters for nanopore as `--max_cluster_bias_INS 100 --diff_ratio_merging_INS 0.3 --max_cluster_bias_DEL 100 --diff_ratio_merging_DEL 0.3`. To get SVs in resolution at single cell level, we set `--min_support 1` to keep all SVs supported by any read of any single cell.

To merge single-cell SV sets by cell line, we used SURVIVOR[26] (v1.0.7). The command called was `SURVIVOR merge $sample_list 500 $c $ident_type $ident_strand $estimate_dist $min_len $out.vcf`. `$sample_list` refers to the path of a text file recording the paths of vcf files to be merged. `500` stands for the maximum base-pair distance to merge two SVs. `$c` set to 1 means that each SV supported by at least one cell would be included. `$ident_type` was set to 1 to enable a check on the agreement of SV type. `$ident_strand` was set to 1 to enable a check on the agreement of strand type. `$estimate_dist` was set to 1 to enable estimation of the distance between SVs. `$min_len` set to 50 means that the minimum size of the reserved SV is 50 bp. Finally, `$out.vcf` is a vcf file that integrates all single-cell SVs of each cell line. It also recorded the counts of cells supporting each SV, which would serve as a proxy of the SV quality later.

We limited the validation of K562 SVs to somatic insertions and deletions. There were two germline SV filters applied to the original SV set. One germline SV filter was the indel variation set called from - scNanoATAC reads of GM12878 that were supported by at least 2 cells. The other filter was based on populational germline SV datasets provided by the Human Genome Structural Variation Consortium[27] (HGSVC) and the Genome Aggregation Database[28] (gnomAD). We removed those known germline SVs from our SV set of K562 and therefore got the somatic SV set for PCR validation. The method for performing the set operation of SVs is described in the next section.

Evaluation of SV calling

Before evaluation of SV calling from scNanoATAC-seq libraries, we made a benchmark SV set ahead. Bulk genomic reads of the K562 cell line sequenced on the ONT platform were used for SV calling. Following the procedures described previously, we aligned raw reads to the reference genome (hg38) directly and used cuteSV to call SV. With the parameter of `--min_support 10` in cuteSV, we only kept SVs supported by at least 10 reads and produced a high-confidence benchmark SV set of K562.

Evaluation of SV calling was entirely performed in the R environment. Firstly, `readVcf` function

of the VariantAnnotation[29] (v1.38.0) package loaded SV calling sets in vcf files into VCF class objects. As SVs are identified from breakpoints of read alignment, we converted original SV records into breakpoints instead to make the determination of the equivalence of two SVs straightforward. This conversion was conducted by `breakpointRanges` function of the StructuralVariantAnnotation[30] (v1.11.0) package with `ignoreUnknownSymbolicAlleles = TRUE`. SVs longer than 50 bp were kept for evaluation. Once both test and benchmark SV sets were converted into breakpoint objects, the intersection between them was calculated by each SV type in units of breakpoints using `countBreakpointOverlaps` function. `maxgap` was set to 500 bp as the error-tolerance for breakpoint comparison. Breakpoints in the test set with at least one overlap with the benchmark set were regarded as validated. We used the numbers of cells supporting each SV as a proxy of SV quality. Then we calculated precision and recall as previously defined under different cut-offs of supporting cell number by SV type. When making Venn diagrams, we counted breakpoints and converted breakpoint numbers into counts of SV events. Two breakpoints represent each insertion, deletion, or duplication event, while four breakpoints represent each inversion event.

Identification of the co-accessible neighboring peak pairs

As the ends of long reads in scNanoATAC-seq stand for Tn5 transposable sites, we can find co-accessible peaks intuitively if two neighboring peaks were linked multiple times by long reads in a specific cell type. To generate a hypothesis test strategy for identifying co-accessible neighboring peak pairs, we assumed that the length of reads supporting a specific peak will be affected if there is a peak neighboring accessible in the same single cell. This alteration of length distribution of peak-supporting reads was detected by Kolmogorov-Smirnov test[31] against the background read length distribution.

In practice, we extracted scNanoATAC-seq read coordinates and made hypothesis tests by cell type. For each peak, we selected reads supporting it by direction, which meant reads with left or right ends inside it were separated. If right ends of long reads fall into a peak, they are potential to support a co-accessibility event at the left side of it, and vice versa. The length distribution of peak supporting reads was derived from those with ends fell into the 1 kb region centered on the target peak, while the null distribution of read length was derived from reads in the 100 kb region around. Two-sided Kolmogorov-Smirnov test of the two distributions was implemented by `ks.test` function in R. The P values were adjusted by `p.adjust` function with `fdr` method, and items with adjusted P value less than 0.05 were kept. Because we tested the potential of co-accessibility in two directions, we can select the neighboring peak pairs with opposite directions of co-accessibility, i.e., the left peak has length alteration of supporting reads at its right side, and vice versa. So far, we identified co-accessible neighboring peak pairs in each cell type. As the co-accessibility was sensed by the alteration of read length, we realized that our detection of co-accessibility cannot exceed the limit of read length. Therefore, we filtered those co-accessible peak pairs with distance greater than twice the median length of peak supporting reads.

Computation of the single-cell CNV matrices

As our CNV analysis was aimed at detecting aneuploidies at single-cell level, while the ploidies are inconsistent among cell lines, we defined the CNV as the fold change of copy number in each bin against the average copy number across the whole genome of each cell line. Therefore, the baseline

of copy number was normalized to 1. In brief, a scNanoATAC-seq coverage matrix was processed into single-cell CNV matrix by binning, copy number normalizing, noise control, smoothing, value clipping, and gap removing:

1) The tile matrix in the ArchR project was re-binned with 100 kb bins by `addTileMatrix` with `tileSize=1e5` and `binarize = false`.
2) We calculated the average fragment number by bin and normalized it to 1 for each single cell.
3) To get control of scNanoATAC-seq, we merged single cells of each cell line by averaging values across cells in each bin. In this way, we generated a pseudo bulk CNV matrix by cell line. Next, we normalized the copy number of pseudo bulk matrix to 1. As 3 out of 5 cell lines we sequenced were euploid, we used the median by bin of the pseudo bulk CNV matrix as an approximate euploid control. The normalized single-cell CNV matrix was controlled by dividing the control vector to eliminate bias in coverage by scNanoATAC-seq. The mean copy number of bins in each single cell was normalized to 1 again after noise control.
4) To smooth the copy number value by bin in each single cell, we exploited `ma` function in the forecast[32] (v8.15) package in R to smooth vectors by moving average. For each bin in single cell, the value was imputed by the average of flanking 100 bins. This operation was applied by chromosome to avoid interference among chromosomes around their boundaries. The copy number values of the first and last 50 bins on each chromosome were missing values, because less than 100 bins around them were available.
5) To make the single-cell CNV matrix less noisy, we clipped the matrix from 0.75 to 1.25 to 1 and limited the maximum of the matrix to 2. Missing values in the matrix were set to 1.
6) Finally, bins intersected with the gaps in genome assembly from the UCSC table browser[33] or the black-list regions given by ArchR were removed from the CNV matrix. The final scNanoATAC-seq CNV matrix was plotted by the pheatmap[34] (v1.0.12) R implementation.

References

1 Chen, X., Miragaia, R. J., Natarajan, K. N. & Teichmann, S. A. A rapid and robust method for single cell chromatin accessibility profiling. *Nat Commun* **9**, 5345, doi:10.1038/s41467-018-07771-0 (2018).

2 McDaniell, R. *et al.* Heritable individual-specific and allele-specific chromatin signatures in humans. *Science* **328**, 235-239, doi:10.1126/science.1184655 (2010).

3 Kumasaka, N., Knights, A. J. & Gaffney, D. J. Fine-mapping cellular QTLs with RASQUAL and ATAC-seq. *Nat Genet* **48**, 206-213, doi:10.1038/ng.3467 (2016).

4 Huddleston, J. *et al.* Discovery and genotyping of structural variation from long-read haploid genome sequence data. *Genome Res* **27**, 677-685, doi:10.1101/gr.214007.116 (2017).

5 Wick, R. R., Judd, L. M. & Holt, K. E. Performance of neural network basecalling tools for Oxford Nanopore sequencing. *Genome Biol* **20**, 129, doi:10.1186/s13059-019-1727-y (2019).

6 Martin, M. Cutadapt removes adapter sequences from high-throughput sequencing reads. *EMBnet.journal* **17**, 10--12, doi:10.14806/ej.17.1.200 (2011).

7 Li, H. Minimap2: pairwise alignment for nucleotide sequences. *Bioinformatics* **34**, 3094-3100, doi:10.1093/bioinformatics/bty191 (2018).

8 Danecek, P. *et al.* Twelve years of SAMtools and BCFtools. *Gigascience* **10**, doi:giab00810.1093/gigascience/giab008 (2021).

9 Quinlan, A. R. & Hall, I. M. BEDTools: a flexible suite of utilities for comparing genomic

features. *Bioinformatics* **26**, 841-842, doi:10.1093/bioinformatics/btq033 (2010).

10 Granja, J. M. *et al.* ArchR is a scalable software package for integrative single-cell chromatin accessibility analysis. *Nat Genet* **53**, 403-411, doi:10.1038/s41588-021-00790-6 (2021).

11 R Core Team. R: A Language and Environment for Statistical Computing. (2021).

12 Zhang, Y. *et al.* Model-based analysis of ChIP-Seq (MACS). *Genome Biology* **9**, doi:10.1186/gb-2008-9-9-r137 (2008).

13 Weirauch, M. T. *et al.* Determination and inference of eukaryotic transcription factor sequence specificity. *Cell* **158**, 1431-1443, doi:10.1016/j.cell.2014.08.009 (2014).

14 Langmead, B. & Salzberg, S. L. Fast gapped-read alignment with Bowtie 2. *Nature Methods* **9**, 357-U354, doi:10.1038/Nmeth.1923 (2012).

15 Moore, J. E. *et al.* Expanded encyclopaedias of DNA elements in the human and mouse genomes. *Nature* **583**, 699-710, doi:10.1038/s41586-020-2493-4 (2020).

16 Lawrence, M. *et al.* Software for computing and annotating genomic ranges. *Plos Computational Biology* **9**, doi:10.1371/journal.pcbi.1003118 (2013).

17 Larsson, J. eulerr: Area-proportional Euler and Venn diagrams with ellipses. R package version 6.1.1. (2021).

18 Zook, J. M. *et al.* Integrating human sequence data sets provides a resource of benchmark SNP and indel genotype calls. *Nat Biotechnol* **32**, 246-251, doi:10.1038/nbt.2835 (2014).

19 Zook, J. M. *et al.* Extensive sequencing of seven human genomes to characterize benchmark reference materials. *Sci Data* **3**, 160025, doi:10.1038/sdata.2016.25 (2016).

20 Patterson, M. *et al.* WHATSHAP: Weighted Haplotype Assembly for Future-Generation Sequencing Reads. *J Comput Biol* **22**, 498-509, doi:10.1089/cmb.2014.0157 (2015).

21 Edge, P., Bafna, V. & Bansal, V. HapCUT2: robust and accurate haplotype assembly for diverse sequencing technologies. *Genome Res* **27**, 801-812, doi:10.1101/gr.213462.116 (2017).

22 Fairley, S., Lowy-Gallego, E., Perry, E. & Flicek, P. The International Genome Sample Resource (IGSR) collection of open human genomic variation resources. *Nucleic Acids Research* **48**, D941-D947, doi:10.1093/nar/gkz836 (2020).

23 Garrison, E. & Marth, G. Haplotype-based variant detection from short-read sequencing. *arXiv [q-bio.GN]* (2012).

24 Benjamini, Y. & Hochberg, Y. Controlling the False Discovery Rate - a Practical and Powerful Approach to Multiple Testing. *J R Stat Soc B* **57**, 289-300, doi:DOI 10.1111/j.2517-6161.1995.tb02031.x (1995).

25 Jiang, T. *et al.* Long-read-based human genomic structural variation detection with cuteSV. *Genome Biology* **21**, doi:10.1186/s13059-020-02107-y (2020).

26 Jeffares, D. C. *et al.* Transient structural variations have strong effects on quantitative traits and reproductive isolation in fission yeast. *Nature Communications* **8**, doi:10.1038/ncomms14061 (2017).

27 Ebert, P. *et al.* Haplotype-resolved diverse human genomes and integrated analysis of structural variation. *Science* **372**, 48-+, doi:10.1126/science.abf7117 (2021).

28 Collins, R. L. *et al.* A structural variation reference for medical and population genetics. *Nature* **581**, 444-+, doi:10.1038/s41586-020-2287-8 (2020).

29 Obenchain, V. *et al.* VariantAnnotation: a Bioconductor package for exploration and annotation of genetic variants. *Bioinformatics* **30**, 2076-2078, doi:10.1093/bioinformatics/btu168 (2014).

30 Cameron, D. & Dong, R. StructuralVariantAnnotation: Variant annotations for structural

variants. R package version 1.11.0. (2021).
31 Massey, F. J. The Kolmogorov-Smirnov Test for Goodness of Fit. *Journal of the American Statistical Association* **46**, 68-78, doi:Doi 10.2307/2280095 (1951).
32 Hyndman, R. J. & Khandakar, Y. Automatic time series forecasting: The forecast package for R. *Journal of Statistical Software* **27**, 1-22, doi:DOI 10.18637/jss.v027.i03 (2008).
33 Karolchik, D. *et al.* The UCSC Table Browser data retrieval tool. *Nucleic Acids Res* **32**, D493-496, doi:10.1093/nar/gkh103 (2004).
34 Kolde, R. pheatmap: Pretty Heatmaps. R package version 1.0.12. (2019).

Protocol for scNanoATAC-seq

Cell preparation

1) 100,000-200,000 cells are pelleted and resuspended with QuickFreezing-M cell freezing medium (Biodragon; KX0310041). The frozen cells can be stored in liquid nitrogen for months before scNanoATAC-seq.

Lysis buffer preparation

2) Prepare the plates (96-well) containing 2 µl 2X Lysis Buffer and 2 µl 5 µM Inner Primer (CTACACGACGCTCTTCCGATCT-[24 bp inner barcode 1-48]-TCGTCGGCAGCGTCAGAT). These plates can be stored at -80 °C.

Recipe for 2X Lysis Buffer:

	Stock	Final con.	Volume (µl)	
Tris-HCI, pH 8.0	1 M	100 mM	0.2	
NaCl	1 M	100 mM	0.2	
Proteinase K	20 mg/ml	40 µg/ml	0.004	
SDS	10%	0.4%	0.08	
H_2O			1.516	
Total			2	

Isolation of nuclei

3) Cells are quickly thawed at 37 °C and centrifuged at 500 × g for 5 min at 4 °C.
4) Pellet cells with 300-500 µl ice-cold Omni-ATAC Lysis Buffer and leave on ice for 3 minutes, and then centrifuge at 800 × g for 5 min at 4 °C.

Recipe for Omni-ATAC Lysis Buffer:

	Stock	Final con.	Volume (µl)	
NP40	10%	0.1%	10	
Tween-20	10%	0.1%	10	
Tris-HCl, pH7.4	1 M	10 mM	10	
NaCl	1 M	10 mM	10	
$MgCl_2$	1 M	3 mM	3	

Digitonin	5%	0.01%	2	
H_2O			955	
Total			1mL	

Tagmentation with Tn5 (embedded with only one adaptor sequence)

5) Resuspend the nuclei pellet with 50 μl tagmentation mix.

Recipe for the tagmentation mix:

	Volume (μl)	
4X THS-seq TD buffer	12.5	
10X Digitonin	5	
H_2O	22.5	
Tn5 (8 μM)	10	
Total	50	

Note: The customized transposase we used with only one adaptor sequence (TCGTCGGCAGCGTCAGATGTGTATAAGAGACAG) was purchased from Novoprotein.

Recipe for 4X THS-seq TD buffer:

	Stock	Final con.	Volume (μl)	
Tris-acetate, pH 7.8	1 M	132 mM		
Potassium acetate	2 M	264 mM		
Magnesium acetate	0.5 M	40 mM		
Dimethylformamide (DMF)	100%	64%		
Total				

Recipe for 10X Digitonin: 1 μl Digitonin (5% stock) + 49 μl H_2O

6) Put the tagmentation reaction (50 μl) on a thermomixer, 37 °C, 800 rpm, 30 minutes.
7) Stop the reaction by adding 50 μl tagmentation stop buffer (TSB). Leave on ice for 10 minutes.

Recipe for TSB:

	Stock	Final con.	Volume (μl)	
Tris-HCI, pH 8.0		10 mM		
EDTA, pH 8.0		20 mM		
Total				

Single nucleus sorting

8) Nuclei are stained with DAPI (1:1k-10k). Single nucleus was flow sorted into each well of 96-well plate containing 4 μL of lysis buffer.

First round PCR

9) Incubate the plate at 65 °C for 30 minutes.
10) Add equal volume (4 μl) of 10% Tween-20 to each well to quench SDS.

11) Add 12 μl the 1st PCR mix to each well.

The 1st PCR mix:

	Volume (μl)	
5X GXL buffer	4	
10mM dNTP	0.4	
GXL polymerase	0.4	
H_2O	7.2	
Total	12	

12) The 96-well plate was then vortexed and thermal cycled as follows: 68 °C for 10 min; 95 °C for 1 min; 16 cycles of 94 °C for 15 s, 63 °C for 30 s, 68 °C for 8 min and finally 68 °C for 5 min.

Second round PCR

13) 5 μL of each of 48 wells with different inner barcodes was pooled and purified with 1X AMPure XP beads (Beckman Coulter; A63882) and eluted into 25 μL of H_2O.
14) The second amplification was carried out in a 50 μL GXL DNA Polymerase system with 1 μM Outer Primer (ATCT-[24 bp outer barcode 73-96]-CTACACGACGCTCTTCCGATCT).

The 2nd PCR mix:

	Volume (μl)	
5X GXL buffer	10	
10mM dNTP	1	
GXL polymerase	1	
H_2O	8	
Outer Primer (10 μM)	5	
Total	25	

15) The incubation program was as follows: 95 °C for 1 min, then 4 cycles of 94 °C for 15 s, 63 °C for 30 s, 68 °C for 8 min and finally 68 °C for 5 min.
16) The amplified products were purified with 1X AMPure XP beads twice.
17) 20 products with different outer barcodes (960 cells in total) were pooled together and sequenced on one Oxford Nanopore PromethION 48 (Oxford Nanopore Technologies; R9.4.1) by Grandomics Biosciences (Beijing, China).
